# Photochemically Triggered, Transient, and Oscillatory Transcription Machineries Guide Temporal Modulation of Fibrinogenesis

**DOI:** 10.1021/jacs.4c16829

**Published:** 2024-12-31

**Authors:** Jiantong Dong, Itamar Willner

**Affiliations:** The Institute of Chemistry, The Hebrew University of Jerusalem, Jerusalem 91904, Israel

## Abstract

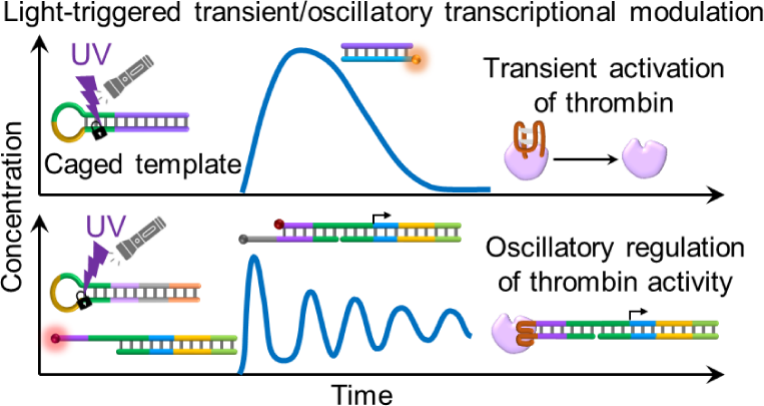

Photochemically triggered, transient, and temporally oscillatory-modulated transcription machineries are introduced. The resulting dynamic transcription circuits are implemented to guide photochemically triggered, transient, and oscillatory modulation of thrombin toward temporal control over fibrinogenesis. One system describes the assembly of a reaction module leading to the photochemically triggered formation of an active transcription machinery that, in the presence of RNase H, guides the transient activation of thrombin toward fibrinogenesis. A second system introduces photochemical triggering of a reaction circuit consisting of two coupled transcription machineries, leading to the temporally oscillatory formation and depletion of an intermediate reaction product. The concept is applied to develop a photochemically triggered transcription circuit that, in the presence of RNase H, leads to the oscillatory generation of an intermediate anti-thrombin aptamer-modified product. The oscillating aptamer-modified product induces the rhythmic inhibition of thrombin, accompanied by the cyclic activation and deactivation of the fibrinogenesis process. The operation of the transient and oscillatory-modulated transcription machinery reaction circuits is accompanied by computational kinetic models, allowing to predict the dynamic behaviors of the system under different auxiliary conditions. The phototriggered transient transcription machinery and oscillatory circuit-guided fibrinogenesis is examined under physiological-like conditions and within a human plasma environment.

## Introduction

Dynamically modulated transcription machineries play key roles in controlling diverse cellular processes, including cell differentiation,^1^ cell cycle progression,^2^ control over intracellular metabolic and physiological balance, and temporal cell development.^3^ These dynamic processes are regulated by transcription factor- or hormone-guided gene expression programs^4−6^ and by auxiliary environmental cues such as pH, stress, or nutrient supply.^7^ Native transcription machineries exhibit diverse dynamic modes, including switchable,^8^ oscillatory,^9,10^ bistable,^11^ and temporal gene expression programs. These gene expression programs are regulated by topological or spatiotemporal control of transcription factor interactions with the transcription circuits, or by proximal or remote regulatory elements,^12−14^ such as enhancers,^15^ silencers,^16^ or insulators.^17^ In particular, native rhythm-dictated processes, such as calcium signaling oscillations,^18^ heartbeat and respiratory rhythms,^19^ hormonal rhythms,^20^ neuronal oscillations,^21^ glycolytic oscillations,^22^ and light/dark-responsive circadian rhythms controlled by molecular clocks that regulate gene expression,^23^ are abundant in nature. This calls for the need to emulate these processes using synthetic circuits that eventually lead to practical applications. Indeed, previous efforts reported on the nucleic acid–based assemblies exhibiting oscillatory activity patterns.^24,25^

Substantial recent research efforts are directed to develop synthetic transcription machineries emulating native systems, and particularly to identify practical applications of these synthetic circuits. Various switchable transcription machineries were reported by introducing reconfigurable blocker units into transcription templates, such as G-quadruplex or triplex units, and gated operation of switchable transcription circuits was demonstrated.^26^ Different strategies for developing transient, dissipative transcription machineries were introduced,^27−29^ including the triggered temporal assembly (activation) and disassembly (deactivation) of transcription templates through the catalytic dissipative operation of transcription processes mediated by DNAzymes or enzymes such as nickase^28,30^ or RNase H.^31^ In addition, bistable transcription circuits,^32−34^ oscillatory transcription circuits,^35,36^ or dynamic transcription clocks^37^ were demonstrated. The applications of dynamic transcription circuits are certainly a key aspect of the topic. Indeed, transcription machineries were employed as functional units for amplified sensing.^38−40^ For example, conjugation of a transcription template to an antigen-modified DNA enabled the amplified detection of antibodies (e.g., anti-digoxigenin antibody),^38^ or the detection of transcription factors (e.g., TetR ligand) by integration of aptamer recognition sequences into the transcription template.^40^ Moreover, applications of transient transcription machineries for temporal release of loads,^41^ transcription-guided temporal biocatalysis (DNAzyme or enzyme activation),^27,31^ and the transient formation and dissociation of DNA nanotube structures^42^ were demonstrated.

The spatiotemporally triggered operation of dynamic transcription machineries is, however, a major challenge, allowing for the fundamental understanding of gene expression pathways and the future controlled and targeted applications of these systems in biological environments. Two different strategies were employed to control the functions of nucleic acids by light. One method has applied photoresponsive caged nucleic acids that are unlocked by light into functional DNA structures, e.g., *ortho*-nitrobenzyl phosphate ester-caging units.^43^ A second approach modified nucleic acids with photoisomerizable units, e.g., trans/cis azobenzene, which controls the stability, configuration, and functions of nucleic acids.^44^ Indeed, photoresponsive caged DNA structures were broadly used for light-stimulated activation of polymerization circuits for diverse sensing applications,^45,46^ light-induced emergence of dynamic DNA assemblies,^47,48^ and light-activated CRISPR/Cas machineries for imaging^49^ and gene editing.^50−52^ Similarly, photoisomerizable units were employed to reconfigure DNA structures, thereby controlling biocatalytic cascades^53^ or switchable catalysis in confined environments,^54^ and dynamic or dissipative DNA networks.^55^ Moreover, light-triggered operation of gene expression pathways is particularly attractive since it provides a rapid ON-OFF switchable, targeted input that eliminates added chemical triggering agents. Indeed, efforts to control gene expression pathways via light using photoresponsive ATP,^56^ nucleic acids,^57,58^ RNA polymerase,^59^ and genetically engineered transcription factors participating in gene expression programs^60^ were reported. For example, T7 RNA polymerase (T7 RNAP) was caged into an inactive, photoresponsive *ortho*-nitrobenzyl carbamate-gated configuration that was switched on by light toward spatiotemporally controlled gene expression.^59,61^ Also, photochemical uncaging of coumarin-protected ATP in the NTPs mixture or *ortho*-nitrobenzyl mismatched oligonucleotides were reported as a means to stimulate controlled RNA polymerization.^56,57^ In addition, photoisomerizable azobenzene-tethered promoter domain of a transcription template provided efficient switchable photoregulation of the transcription process.^62^

Nonetheless, light-stimulated transient transcription machineries or temporally oscillatory-modulated transient transcription machineries are unprecedented. In the present study, we introduce *ortho*-nitrobenzyl phosphate ester-protected hairpin structures that, upon photochemical uncaging in the presence of promoter strands, lead to the emergence of either transient, dissipative transcription machineries or oscillatory-modulated transient transcription circuits. We provide quantitative kinetic models that allow prediction of the dynamic behaviors of the transient transcription reaction modules and the temporally oscillatory-modulated circuits under variable auxiliary conditions. Moreover, beyond the fundamental significance of these complex dynamic systems in demonstrating the phototriggered transient oscillatory activity of transcription machineries, these concepts are applied to develop phototriggered, transcription machinery-guided, transient oscillatory thrombin-catalyzed coagulation of fibrinogen to fibrin. The temporally controlled dynamic thrombin-mediated fibrinogenesis attracts growing interest.^63^ Indeed, several recent studies reported on DNA circuits temporally modulating fibrinogenesis.^31,48,64^ Nevertheless, none of these systems demonstrated the transient oscillatory fibrinogenesis or the capacity to control the amplitude and rhythm by auxiliary agents. Furthermore, we demonstrate that the phototriggered, transcription circuit-guided, transient and oscillatory fibrinogenesis process operates under physiological-like perturbing environments, suggesting potential means for controlling blood clotting.

## Results and Discussion

### Phototriggered Dissipative Transcription Machineries Guiding Transient Activities of Fibrinogenesis

**Figure 1:**
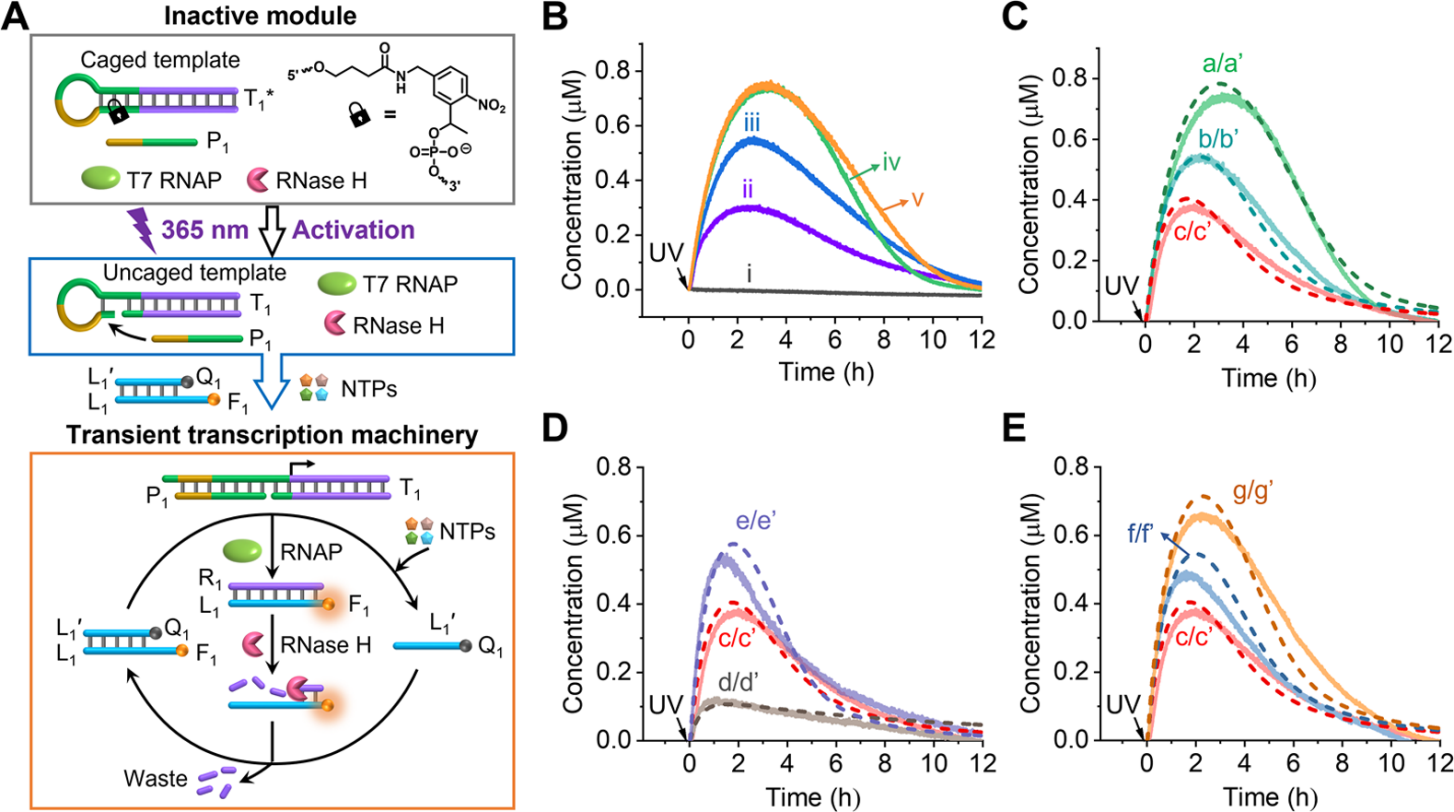
(A) Schematic of the reaction module presenting the light-triggered activation of a transient transcription machinery. The temporal, transient operation of the reaction module is probed by the time-dependent fluorescence intensities of the fluorophore (F_1_ = Cy3)-labeled R_1_/L_1_. (B) Temporal concentration changes of the intermediate R_1_/L_1_ generated using different illumination time-intervals: (i) 0, (ii) 1, (iii) 2, (iv) 6, and (v) 10 min. (C) Transient concentration changes of R_1_/L_1_ following 6 min of light activation in the presence of variable concentrations of RNase H: (a/a′) 0.20 nM, (b/b′) 0.27 nM, and (c/c′) 0.33 nM. Solid curves a, b, and c represent experimental results, and dashed curves a′, b′, and c′ are computationally simulated and predicted curves. Other experimental conditions: T_1_* = 0.2 μM, P_1_ = 0.2 μM, L_1_/L_1_′ = 1.6 μM, T7 RNAP = 48 nM, NTPs = 0.5 mM, 35 °C. (D) Transient concentration changes of R_1_/L_1_ corresponding to the light-triggered dissipative transcription machineries in the presence of different concentrations of T7 RNAP: (c/c′) 48 nM, (d/d′) 16 nM, and (e/e′) 64 nM. (E) Transient concentration changes of R_1_/L_1_ upon subjecting the light-triggered reaction module to variable concentrations of NTPs: (c/c′) 0.5 mM, (f/f′) 0.6 mM, and (g/g′) 0.7 mM. Experimental conditions for the curves shown in (D) and (E) are similar to those described for curve c/c′.

In the first step, a photochemically triggered dissipative transient machinery was assembled, Figure 1A. The reaction module consists of an *ortho*-nitrobenzyl phosphate ester-caged photoresponsive hairpin framework T_1_*, a promoter strand P_1_, T7 RNAP, and RNase H. The reaction module exists in an inactive configuration. Photochemical cleavage of the caging units under UV illumination (λ = 365 nm) results in the promoter (P_1_)-induced displacement of the cleaved hairpin domain to yield an active transcription template P_1_/T_1_. A fluorophore F_1_ (Cy3)/quencher Q_1_ (BHQ2)-modified duplex L_1_/L_1_′, acting as an auxiliary reporter unit to transduce the dynamic operation of the reaction module, and NTPs are added to the system immediately after photochemical uncaging of template T_1_*. (The reason for adding the reporter unit after the photochemical uncaging is to avoid the partial bleaching of the fluorophore caused by the illumination process.) Under these conditions, the T7 RNAP/NTPs transcription machinery is activated, yielding the RNA product R_1_. The R_1_ product is pre-engineered to displace the duplex L_1_/L_1_′, resulting in the formation of the displaced quencher strand L_1_′ and the fluorescent R_1_/L_1_ duplex. The competitive cleavage of R_1_ by RNase H leads, however, to the reverse binding of L_1_′ to L_1_, generating the parent fluorescence-quenched duplex L_1_/L_1_′. That is, the light-triggered cleavage of template T_1_* activates a transient dissipative process generating the fluorescent intermediate R_1_/L_1_, where the transient fluorescence intensities of F_1_ linked to L_1_ serve as the readout signal for this process. (Control experiments demonstrating the photochemical uncaging of T_1_* and the activation of the transcription machinery were performed using gel electrophoresis and fluorescence as readout signals, Figure S1.) Using an appropriate calibration curve relating the fluorescence intensity of F_1_–L_1_ to its concentrations (Figure S2), the temporal concentrations of the intermediate R_1_/L_1_ are controlled by the dose (time) of photocleavage of T_1_*, Figure 1B. As the photocleavage (λ = 365 nm, 20 mW/cm^2^) is prolonged, the peak concentration of the intermediate product increases and reaches a saturation level after 6 min (Figure S3). Accordingly, in all subsequent experiments, the illumination time required to yield the active transcription machinery was selected to be 6 min. Figure 1C, curve a, depicts the transient, temporal concentration of the intermediate R_1_/L_1_ in the presence of 6 U/mL (0.20 nM) RNase H, 3 U/μL (48 nM) T7 RNAP, and 0.5 mM NTPs. A kinetic model for the dynamic circuit was formulated (Figures S4–S6 and accompanying discussion). The simulated fitting results are presented in Figure 1C, curve a′ (dashed curve), and the corresponding rate constants are summarized in Table S1. The dynamic temporal formulation and depletion of the intermediate R_1_/L_1_ are anticipated to be controlled by the concentrations of RNase H, T7 RNAP, and NTPs. Accordingly, the kinetic model and derived rate constants were adopted to predict the temporal behavior of R_1_/L_1_ in the presence of variable concentrations of RNase H, T7 RNAP, and NTPs, and the predicted transient results were validated experimentally. Figure 1C compares the kinetically predicted temporal formation and depletion of the intermediate R_1_/L_1_ at varying concentrations of RNase H (curves b′ and c′ in dashed lines) with the corresponding experimental results in the presence of these RNase H concentrations (curves b and c in solid curves). The experimental results fit well to the computationally predicted behaviors of the system. Moreover, Figure 1D depicts the predicted (dashed lines) and experimentally validated results (solid curves), d/d′, c/c′, e/e′, corresponding to the formation/depletion of R_1_/L_1_, in the presence of variable concentrations of T7 RNAP. Additionally, Figure 1E presents the predicted and experimental transient curves corresponding to the formation and depletion of the intermediate R_1_/L_1_ at varying concentrations of NTPs. The experimental results fit well to the computationally predicted outcomes, supporting the value of the kinetic model and the derived rate constants as effective tools for predicting the dynamic behaviors of the reaction module under different auxiliary conditions. (The peak concentration values and the time intervals for the emergence of these peak concentrations in the presence of variable concentrations of RNase H, T7 RNAP, and NTPs are provided in Figure S7.)

**Figure 2:**
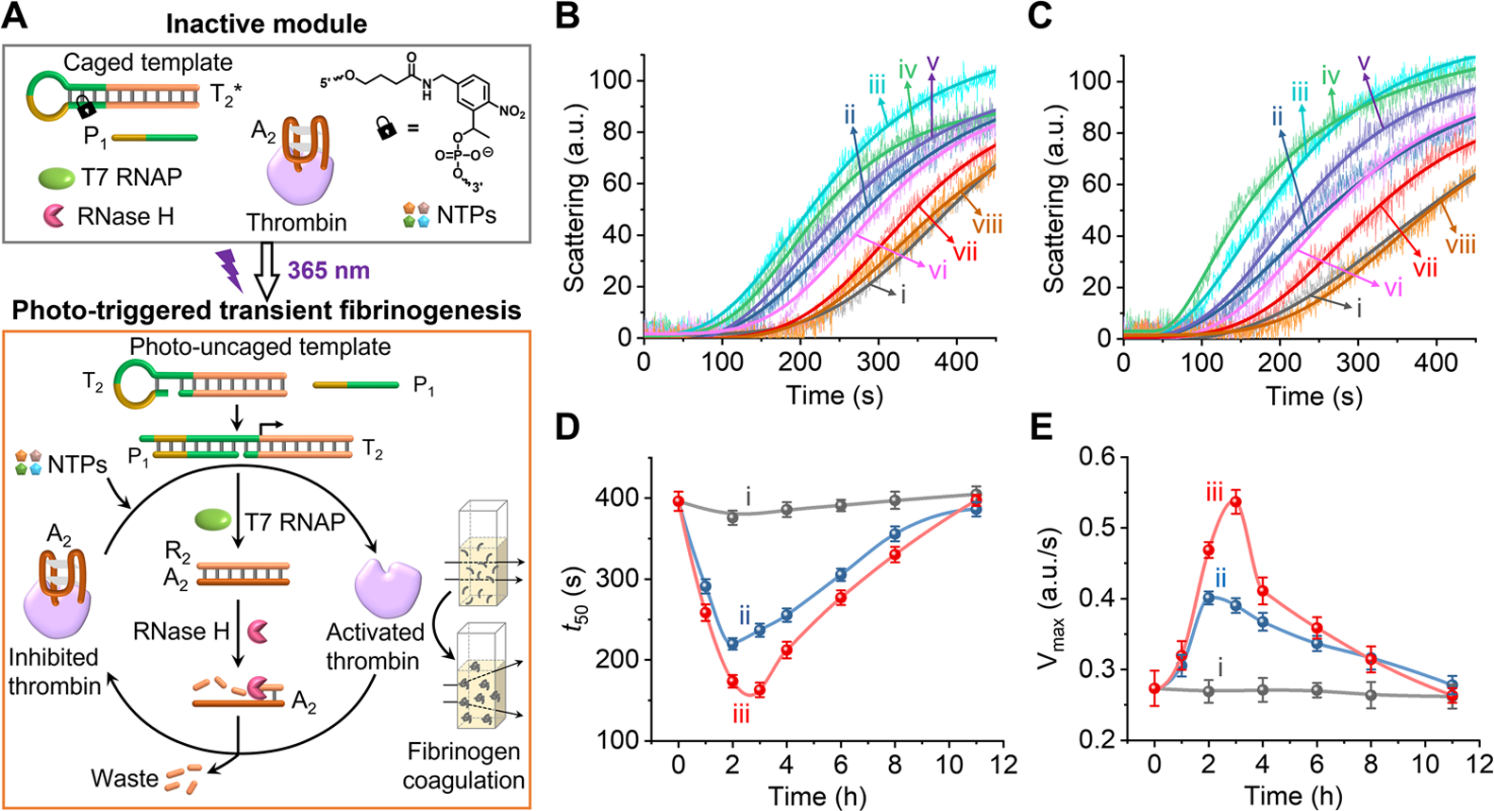
(A) Schematic configuration and operation of a photoactivated transcription machinery guiding the transient fibrinogenesis through the temporal, transient activation of thrombin. (B) Dynamic light-scattering kinetic profiles generated by samples withdrawn at time intervals from the reaction module shown in (A) after photochemical uncaging for 2 min (λ = 365 nm, 20 mW/cm^2^): (i) 0, (ii) 1, (iii) 2, (iv) 3, (v) 4, (vi) 6, (vii) 8, and (viii) 11 h. (C) Temporal light-scattering kinetic profiles generated by samples withdrawn at time intervals from the reaction module after photochemical uncaging for 6 min: (i) 0, (ii) 1, (iii) 2, (iv) 3, (v) 4, (vi) 6, (vii) 8, and (viii) 11 h. (D) Temporal *t*_50_ values obtained from analyzing the dynamic light-scattering curves corresponding to (i) a caged reaction module without photochemical activation (results presented in Figure S8), (ii) the reaction module after photochemical uncaging for 2 min (results presented in (B)), (iii) the reaction module after photochemical uncaging for 6 min (results presented in (C)). (E) Transient temporal *V*_max_ values derived from the dynamic light scattering curves corresponding to (i) the caged reaction module without photochemical activation, (ii) the reaction module after photochemical uncaging for 2 min, (iii) the reaction module after photochemically uncaged for 6 min.

The visionary applications of dynamic networks and circuits are a major challenge in the field. We identified the thrombin-catalyzed transformation of fibrinogen to fibrin, a key process in blood coagulation, as a target for temporal regulation by dynamic DNA networks. That is, the temporal, dose-controlled regulation of blood clotting could find important future medical uses of nanostructured DNA circuits. Figure 2A depicts the schematic of the light-triggered, transcription machinery-guided, transient, and temporal activation of the thrombin-catalyzed transformation of fibrinogen to fibrin. The inactive reaction module includes an *ortho*-nitrobenzyl phosphate ester-caged, photoresponsive hairpin template T_2_*, a promoter strand P_1_, the enzymes T7 RNAP and RNase H, NTPs, and thrombin inhibited by the anti-thrombin aptamer A_2_. Photochemical deprotection of the template through photocleavage of the hairpin, followed by hybridization of the promoter to the cleaved, uncaged template, yields the active transcription machinery P_1_/T_2_. The template T_2_ was designed, however, to yield the RNA R_2_ sequence, which is complementary to the A_2_ aptamer that inhibits thrombin. Displacement of A_2_ from the A_2_/thrombin complex, through the formation of the R_2_/A_2_ complex, yields active thrombin for the biocatalyzed transformation of fibrinogen to fibrin. The concomitant RNase-mediated cleavage of R_2_ within the RNA/DNA (R_2_/A_2_) duplex releases A_2_, which rebinds to thrombin, leading to its inhibition. That is, the light-triggered deprotection of the reaction module activates the transcription machinery, guiding the temporal activation and depletion of free thrombin as an intermediate functional agent catalyzing the transient transformation of fibrinogen to fibrin. The dynamic control over the temporal functions of thrombin is then followed by the transient dynamic light-scattering features of the reaction mixture. Figure 2B depicts the dynamic light-scattering intensities associated with samples withdrawn at time intervals from the reaction module, which was activated by the light-induced uncaging of the transcription template for 2 min. Evidently, the dynamic light-scattering curves, reflecting the coagulation rates, show an initial enhancement for the first 2 h, followed by a gradual retardation over the next 8 h, eventually returning to the baseline light-scattering characteristics of inhibited thrombin. In addition, Figure 2C shows the temporal light-scattering kinetic profiles associated with the thrombin-catalyzed coagulation of fibrinogen to fibrin in samples withdrawn from the transcription machinery reaction mixture after activation by the light-triggered uncaging of the transcription template for 6 min. A control study probing the temporal light-scattering kinetic profiles of non-illuminated reaction samples did not show any changes in the light-scattering rate, compared to the background output of the parent inactive reaction module, Figure S8. This indicates that the dynamic light-scattering kinetic profiles observed in Figure 2B,C indeed originate from the light-triggered transcription machinery-guided control over the temporal activation and deactivation of thrombin-catalyzed fibrinogenesis. Figure 2D,E display the analysis of the temporal light-scattering kinetic curves using two presentation methods, highlighting the transient, dissipative features of the fibrinogenesis process. Taking the time interval reaching the threshold of light-scattering intensity (50 au) as a quantitative value (*t*_50_) for assessing the efficacy of fibrinogenesis, the time-dependent changes in *t*_50_ values reflect the temporal kinetics of the fibrinogenesis process and the temporal activities of thrombin. Figure 2D presents the temporal values of *t*_50_ for the reaction module activated by different UV illumination time-intervals. The fueled activation of the reaction module leads to a decreased *t*_50_ value after 2 h, indicating the uncaging of thrombin and the resulting rapid fibrinogenesis process. At longer time intervals, the temporal *t*_50_ values increase, and after a time interval of 8 h, the high level of *t*_50_ characterizing the rest module is recovered. With a longer illumination time (6 min), the minimum value of *t*_50_ is lower and appears later. In addition, the maximum coagulation rates (*V*_max_) of the light-scattering kinetic profiles are related to the catalytic activities of the uncaged thrombin. Figure 2E presents the temporal values of *V*_max_ upon activation of the system with different UV illumination time intervals. While the *V*_max_ value characterizing the rest module is very low, consistent with the aptamer-inhibited thrombin, light-triggered activation of the transient transcription machinery yields, after 2 h, an uncaged active thrombin that leads to effective fibrinogenesis. This is followed by a temporal deactivation of thrombin for about 8 h, resulting from the RNase H depletion of the antidote R_2_ and the recovery of the inhibited aptamer-thrombin complex. Evidently, as the time interval of light-triggered uncaging of the inactive template T_2_* is prolonged, the peak *V*_max_ increases, due to the higher content of uncaged transcription template.

### Phototriggered Transient Oscillatory Transcription Circuits Guiding Oscillatory Fibrinogenesis

**Figure 3:**
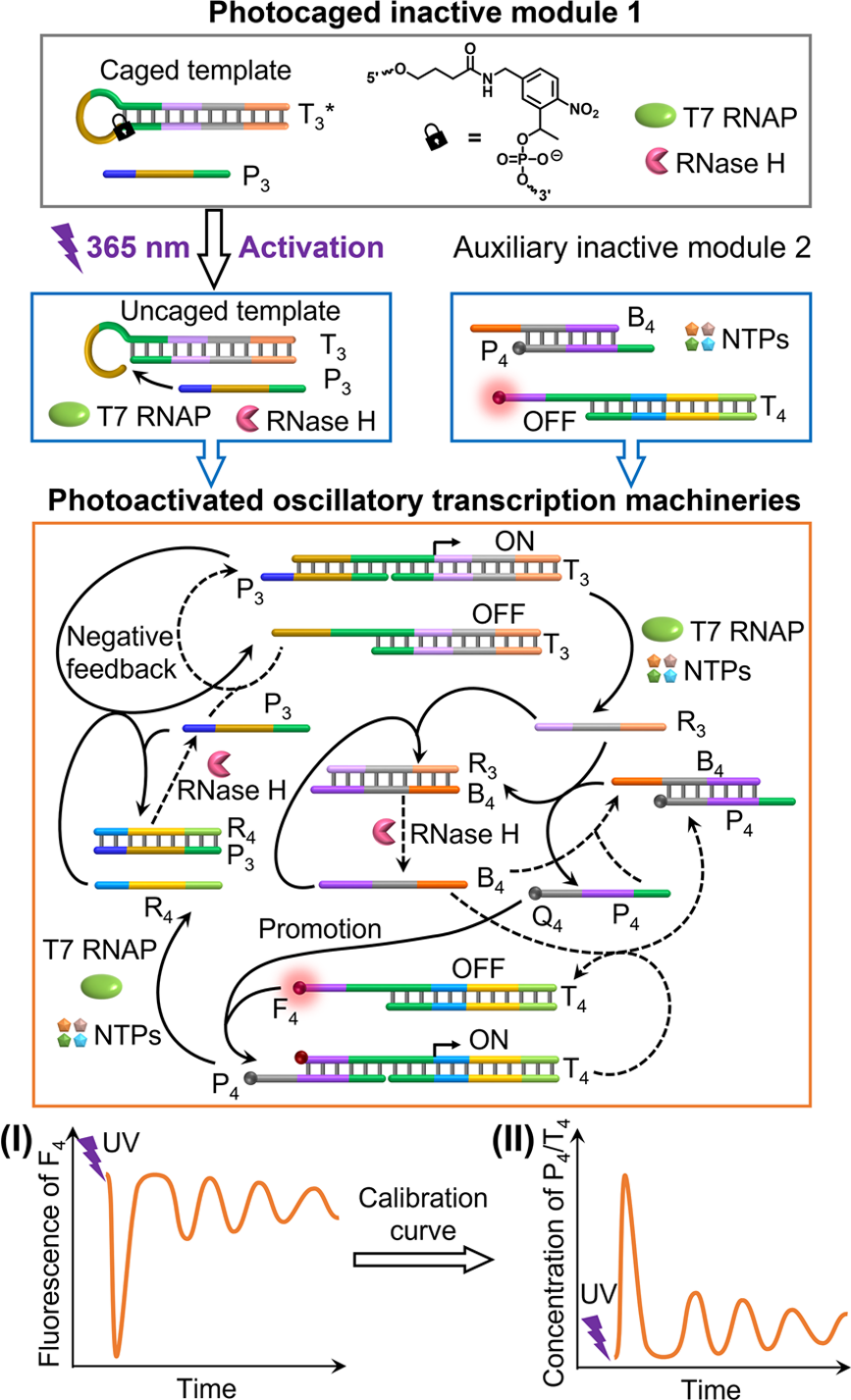
Schematic representation of the photochemically triggered operation of a transient oscillatory-modulated transcription circuit, consisting of two coupled transcription machineries. The oscillatory operation of the circuit is transduced by the temporal fluorescence intensities of constituent T_4_ (panel I) and converted into the corresponding temporal concentrations of P_4_/T_4_ (panel II) using an appropriate calibration curve.

The temporal modulation of transient dissipative circuits, and particullary the identification of possible applications of temporally modulated transcription machineries, could add further complexity to the field of disspative systems and signifcantly enhance the programmability of such reaction modules. Following these concepts, we developed a light-triggered oscillatory transcription machinery and adopted these principles to guide the temporal modulation of fibrinogenesis. The assembly and operation of the photoactivated oscillatory transcription circuit are schematically displayed in Figure 3. The reaction module consists of the inactive *ortho*-nitrobenzyl phosphate ester-caged hairpin template T_3_*, a promoter strand P_3_, T7 RNAP, and RNase H. After photochemical uncaging of the template T_3_*, an inactive template T_4_ consisting of a fluorophore (TexasRed)-modified duplex and an auxiliary quencher (Iowa Black RQ)-modified duplex P_4_/B_4_, along with NTPs, are added to the reaction module to activate the oscillatory-modulated operation of the transcription machineries. (The sequential integration of the composite circuit is essential to prevent UV light-induced bleaching of the fluorophore-modified template T_4_ and to avoid perturbing the operation of the transcription machinery T_3_/T7 RNAP.) Under these conditions, the oscillatory-modulated transient operation of the composite transcription machineries proceeds. The photo-uncaged template T_3_ is hybridized with the promoter strand P_4_ to form an active promoter-linked template P_4_/T_3_. In the presence of T7 RNAP and NTPs, the transcription machinery yields the RNA product R_3_. The transcribed R_3_ displaces the DNA duplex P_4_/B_4_, forming the duplex R_3_/B_4_ and releasing the quencher-labeled strand P_4_. The released quencher-labeled strand P_4_ acts as a promoter that binds to the fluorophore-labeled template T_4_, activating the second transcription machinery T_4_ and generating the RNA product R_4_. R_4_, in turn, displaces and hybridizes the promoter strand P_3_ that initially activated the parent template T_3_, leading to the temporal inhibition of transcription machinery T_3_. RNase H present in the system degrades the RNA strand R_3_ associated with the R_3_/B_4_ duplex, causing the released B_4_ strand to displace P_4_ from the template P_4_/T_4_ and thereby temporarily inhibiting the transcription machinery T_4_. Concomitantly, RNase H cleaves the RNA strand R_4_ in the R_4_/P_3_ duplex, releasing P_3_ which reactivates the transcription machinery T_3_ producing R_3_. That is, the light-triggered activation of the reaction module leads to the transient oscillatory-modulated operation of the coupled transcription machineries T_3_ and T_4_. While the P_3_-driven operation of transcription machinery T_3_ produces R_3_, which promotes the P_4_-driven operation of transcription machinery T_4_, the generated R_4_ provides a negative feedback loop that inhibits transcription machinery T_3_. The oscillatory-modulated operation of the reaction module is regulated by the concomitant cleavage of R_3_ in the R_3_/B_4_ duplex and R_4_ in the R_4_/P_3_ duplex. This transient oscillatory-modulated process proceeds as long as the NTPs fuel is available, generating the fragmented R_3_ and R_4_ as waste products. The temporally oscillatory-modulated transient operation of the reaction circuit is monitored by following the fluorescence features of the template T_4_. In the “rest” configuration of the circuit, the fluorescence of T_4_ is switched on. Phototriggered activation of transcription machinery T_3_ yields R_3_, which promotes the binding of the quencher (Iowa Black RQ)-labeled P_4_ to the template T_4_, thereby quenching the fluorescence of the fluorophore (TexasRed)-modified template. The RNase H-guided concomitant cleavage of R_3_ in the R_3_/B_4_ duplex releases B_4_, temporarily separating P_4_ from the template T_4_ and leading to the recovery of fluorescence. Figure 3, panel I schematically depicts the expected dissipative oscillatory-modulated fluorescence intensities transduced by the reaction module. Using an appropriate calibration curve that relates the decrease in fluorescence intensities to the concentrations of P_4_/T_4_ upon adding different concentrations of P_4_ to quench the fluorescence of T_4_ (Figure S9), the oscillatory concentrations of the activated template P_4_/T_4_ are evaluated, Figure 3, panel II. Evidently, the rhythms of the temporally oscillatory-modulated fluorescence/concentration changes, in terms of oscillation amplitudes, temporal spacing, and transient duration, are anticipated to be controlled by the time interval of UV illumination used to uncage T_3_* into the active P_3_/T_3_, as well as the concentrations of T_3_*, T7 RNAP, RNase H, and the auxiliary strands B_4_ and P_3_.

**Figure 4:**
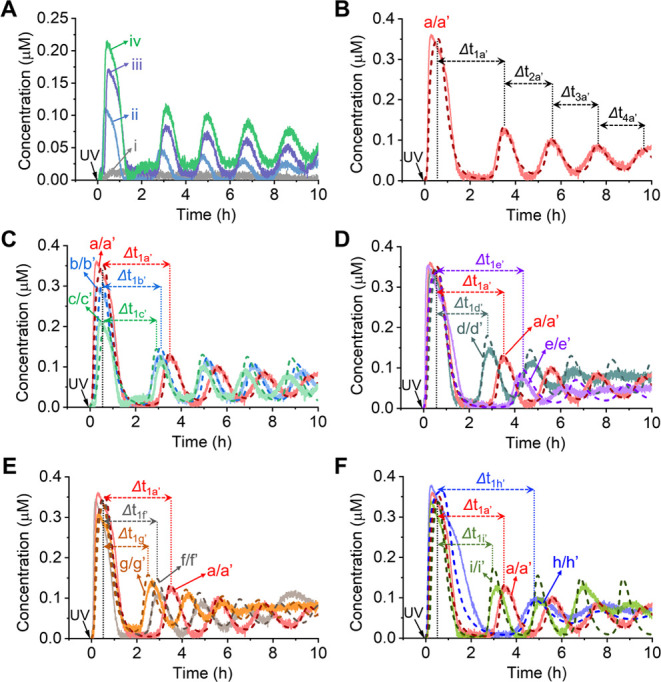
(A) Oscillatory-modulated concentrations of P_4_/T_4_ generated by the transcription circuit shown in Figure 3, resulting from the photochemical uncaging of template T_3_* (70 nM) with UV illumination for different time intervals: (i) 0, (ii) 0.5, (iii) 2, and (iv) 6 min. Experimental conditions: T_3_* = 70 nM, P_3_ = 1.5 μM, B_4_ = 1.5 μM, P_4_ = 0.5 μM, T_4_ = 0.5 μM, T7 RNAP = 128 nM, RNase H = 1.00 nM, and NTPs = 7.5 mM at 35 °C. (B) Oscillatory transient modulated concentrations of P_4_/T_4_ (solid line a) generated upon photochemical deprotection of T_3_* (150 nM) in the circuit for 6 min. The experimental result is modeled and fitted by the computational kinetic model (Figure S11), dashed line a′. Experimental conditions for curve a: T_3_* = 150 nM, P_3_ = 1.5 μM, B_4_ = 1.5 μM, P_4_ = 0.5 μM, T_4_ = 0.5 μM, T7 RNAP = 128 nM, RNase H = 1.00 nM, and NTPs = 7.5 mM at 35 °C. (C) Temporally oscillatory-modulated concentrations of P_4_/T_4_ generated by the circuit following photochemical deprotection of different concentrations of template T_3_* for 6 min: (a/a′) 150 nM, (b/b′) 100 nM, and (c/c′) 70 nM. (D) Temporally oscillatory-modulated concentrations of P_4_/T_4_ following photochemical uncaging of T_3_* (150 nM) for 6 min with different concentrations of T7 RNAP: (a/a′) 128 nM, (d/d′) 96 nM, and (e/e′) 160 nM. (E) Temporally oscillatory-modulated concentrations of P_4_/T_4_ generated upon photochemical uncaging of T_3_* (150 nM) for 6 min in the presence of different concentrations of RNase H: (a/a′) 1.00 nM, (f/f′) 1.16 nM, and (g/g′) 1.33 nM. (F) Temporally oscillatory-modulated concentrations of P_4_/T_4_ generated by the circuit in the presence of different concentrations of B_4_: (a/a′) 1.5 μM, (h/h′) 1.0 μM, and (i/i′) 2.0 μM. Curves b′–i′ shown in (C–F) are computationally predicted curves, and curves b–i represent experimentally validated results. Experimental conditions for these curves are similar to those of curve a.

Figure 4A depicts the oscillatory-modulated operation of the reaction module shown in Figure 3, subjected to different time intervals of UV light uncaging of the protected template T_3_*. As the time intervals for photochemical uncaging of T_3_* increase, the amplitude corresponding to the concentration of the intermediate activated transcription machinery P_4_/T_4_ also increases. The amplitude of P_4_/T_4_ concentration intensifies within the first 30 min and subsequently decays, developing a repeated, oscillatory-modulated pattern that gradually decreases in amplitude over approximately 10 h. These results reflect the transient depletion of the reaction module. The rhythm separating the oscillating peaks, under the specific conditions, corresponds to ca. 2 h. After oscillation time intervals of 10 h, the oscillation amplitudes are slightly distorted, leveling off to a very low nonzero concentration of the intermediate P_4_/T_4_, Figure S10. This may originate from the accumulation of RNA fragments (waste products of R_3_ and R_4_) due to RNase H degradation, which perturbs the oscillatory process by binding to the constituents of the module. Accordingly, subsequent analyses of the oscillatory reaction module, under different conditions, were conducted within a 10-h time frame. Figure 4B, solid curve a, shows the oscillatory-modulated pattern of the reaction intermediate P_4_/T_4_ when the reaction module is operated by photochemically uncaging for a fixed time interval of 6 min, at experimental conditions corresponding to 150 nM T_3_*, 1.5 μM P_3_, 1.5 μM B_4_, 0.5 μM P_4_, 0.5 μM T_4_, 128 nM T7 RNAP, 1.00 nM RNase H, and 7.5 mM NTPs. As a first step to characterize the system, we formulated a kinetic model with an attempt to use the kinetic model and the derived rate constants to predict the oscillatory patterns of the systems under different compositional conditions, which were subsequently validated by experiments. Figure S11 summarizes the set of rate equations describing the stepwise reactions associated with the oscillatory operation of the circuit. Figure 4B, dashed line a′, presents the best fit of the computationally predicted oscillatory concentrations of P_4_/T_4_ using the kinetic model outlined in Figure S11. The computational curve a′ shows a time gap between the first and second oscillating peaks (Δ*t*_1_) corresponding to 3 h and a constant rhythm separating the subsequent oscillating peaks (Δ*t_n_*, *n* ≥ 2) corresponding to 2 h. The set of rate constants associated with the fitted computational curve is summarized in Table S2. These rate constants were used to predict the behavior of the system under different compositional conditions, followed by experimental probing of the reaction module′s operation under these conditions. Figure 4C shows the kinetic model-predicted temporally oscillatory-modulated concentrations of the intermediate P_4_/T_4_ for different concentrations of caged T_3_* being unlocked by light-triggered cleavage for 6 min (curves a′ 150 nM, b′ 100 nM, c′ 70 nM). The predicted patterns indicate that as the concentration of T_3_* decreases, the amplitude of P_4_/T_4_ also decreases. The first oscillation evolves at slightly shorter time intervals, and the time gap between the first and second oscillating peaks is slightly shorter (Δ*t*_1a′_ = 3.0 h, Δ*t*_1b′_ = 2.6 h, Δ*t*_1c′_ = 2.2 h), while the rhythm between the subsequent oscillating peaks (Δ*t_n_*, *n* ≥ 2) remains constant (ca. 2 h). The experimental results overlay these predicted oscillatory patterns. Figure 4D depicts the computationally predicted transient oscillatory-modulated concentrations of the intermediate P_4_/T_4_ for different concentrations of T7 RNAP (curves d′ 96 nM, a′ 128 nM, e′ 160 nM), while retaining the concentrations of other constituents the same as those in curve a′. The predicted patterns indicate that the amplitudes of the first peak remain identical different T7 RNAP concentrations, while the amplitudes of the subsequent oscillating peaks (*n* ≥ 2) decrease as the T7 RNAP concentration increases. The time gap between the first and second oscillating peaks becomes longer (Δ*t*_1d′_ = 2.3 h, Δ*t*_1a′_ = 3.0 h, Δ*t*_1e′_ = 3.7 h), while the rhythm between subsequent oscillating peaks (Δ*t_n_*, *n* ≥ 2) is identical. The experimental results fit well with the predicted oscillatory patterns. Moreover, Figure 4E depicts the computationally predicted transient oscillatory-modulated concentrations of the intermediate P_4_/T_4_ upon subjecting the reaction module to different concentrations of RNase H (curves a′ 1.00 nM, f′ 1.16 nM, g′ 1.33 nM), while the concentrations of other constituents are same as those in curve a′. The predicted results indicate that variable RNase H concentrations have minute effects on the temporally modulated amplitudes. However, as the RNase H concentration increases, the time gap between the first and second oscillating peaks becomes shorter (Δ*t*_1a′_ = 3.0 h, Δ*t*_1f′_ = 2.5 h, Δ*t*_1g′_ = 2.0 h), and the rhythm separating the subsequent oscillating peaks (Δ*t*_*n*_, *n* ≥ 2) is also slightly shortened. The experimental results are consistent with the predicted oscillatory patterns. Furthermore, the effect of different concentrations of the B_4_ constituent, which blocks P_4_ in the rest reaction module, on the temporally modulated process, is presented in Figure 4F. The computationally predicted amplitudes of the intermediate P_4_/T_4_, in the presence of different concentrations of B_4_ (curves h′ 1.0 μM, a′ 1.5 μM, i′ 2.0 μM), are almost identical. However, the time gaps between the first and second oscillating peaks are shorter (Δ*t*_1h′_ = 4.0 h, Δ*t*_1a′_ = 3.0 h, Δ*t*_1i′_ = 2.5 h) as the concentration of B_4_ increases. These results are detailed further in Figure S12. The experimental results the predicted oscillatory patterns. In addition, we examined the effect of different concentrations of P_3_ on the temporally modulated oscillatory patterns of P_4_/T_4_. Computational predictions suggest minimal impact on the amplitudes and rhythms of the oscillating peaks, Figure S13. Indeed, experimental results fit well to these computationally predicted patterns. The results presented in Figure 4 emphasize the effectiveness of the kinetic model in predicting the temporal behavior of the complex oscillatory reaction circuit. The experimental and computational results displayed in Figure 4 indicate that altering individual parameters of the phototriggered transient oscillatory machinery including the concentrations of T_3_*, T7 RNAP, RNase H, and the blocker unit B_4_, affect the amplitudes and time gaps between the oscillation bands. Moreover, Figure S14 demonstrates that the simultaneous change of two parameters, e.g., RNase H and B_4_, further affects the amplitudes and time gaps between the oscillation peaks, and the experimental results are well suggested by the simulations.

**Figure 5:**
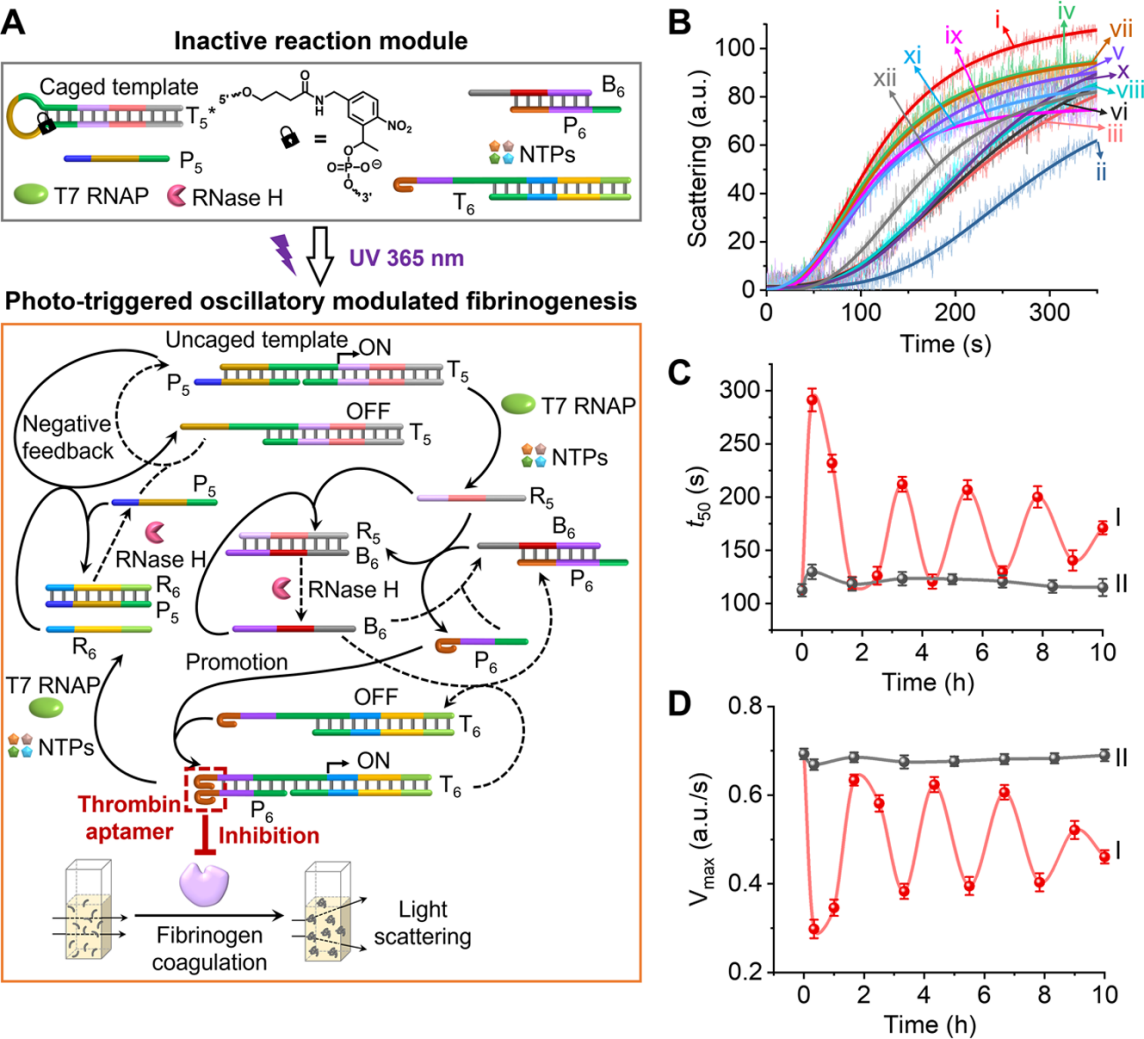
(A) Schematic operation of a phototriggered transcription circuit consisting of two coupled transcription machineries, resulting in the oscillatory regulated fibrinogenesis through the temporally oscillatory assembly and disassembly of an anti-thrombin aptamer unit. (B) Temporal light-scattering kinetic profiles corresponding to the fibrinogenesis processes in samples withdrawn from the reaction mixture described in (A), photochemically uncaged for 6 min, at time intervals: (i) 0, (ii) 20, (iii) 60, (iv) 100, (v) 150, (vi) 200, (vii) 260, (viii) 330, (ix) 400, (x) 470, (xi) 540, and (xii) 600 min. (C) Temporally oscillatory-modulated *t*_50_ values derived from the dynamic light-scattering curves corresponding to (I) results depicted in (B) for the reaction mixture photodeprotected for 6 min, and (II) results derived from the control system presented in Figure S15 for the non-photochemically deprotected reaction mixture. (D) Temporally oscillatory-modulated *V*_max_ values derived from the dynamic light-scattering curves corresponding to (I) results in (B) for the reaction mixture photo-deprotected for 6 min, and (II) results derived from the control system in Figure S15 for the non-photochemically deprotected reaction mixture.

The search for potential useful applications of the photoactivated oscillatory-modulated transcription machineries is certainly a challenge. In the first part of the study, we explored using light-triggered transient transcription machinery as a tool guiding the light-triggered temporal fibrinogenesis through transient activation of thrombin. It seems, however, feasible to adopt the photoactivated, temporally oscillatory-modulated transcription machineries to design a phototriggered temporally oscillatory-modulated fibrinogenesis process through the oscillatory modulation of thrombin. It should be noted that such oscillatory fibrinogenesis programs are unprecedented, and developing such systems could offer significant medical value by introducing temporally controlled blood clotting pathways. Figure 5A depicts the assembly and operation of a photocaged reaction module consisting of coupled transcription machineries that guide phototriggered, temporally oscillatory-modulated fibrinogenesis through the temporally oscillatory inhibition of thrombin. The reaction module comprises the *ortho*-nitrobenzyl phosphate ester-caged hairpin framework T_5_* and the promoter strand P_5_. The inactive transcription template T_6_, T7 RNAP, RNase H, and the auxiliary duplex B_6_/P_6_ are also included in the reaction circuit. Note that the inactive template T_6_ features a domain corresponding to a G-rich subunit of the anti-thrombin aptamer (orange), while the strand P_6_ in the duplex B_6_/P_6_ contains a second subunit of the anti-thrombin aptamer (orange) in a locked configuration. The reaction steps involved in the temporal, oscillatory-modulated regulation of thrombin is displayed in Figure 5A. Photochemical cleavage of the photoresponsive hairpin structure (λ = 365 nm), in the presence of NTPs, initiates the P_5_-stimulated displacement of the cleaved hairpin, yielding the active transcription machinery P_5_/T_5_, which synthesizes the RNA product R_5_. The R_5_ displaces the B_6_/P_6_ duplex, generating the R_5_/B_6_ duplex and releasing P_6_. P_6_ acts as a promoter strand, activating the transcription machinery T_6_. The formation of the active transcription template P_6_/T_6_ involves the assembly of the G-quadruplex anti-aptamer structure (orange) which exhibits the capacity to inhibit thrombin. The P_6_-triggered operation of transcription machinery T_6_ results in the T7 RNAP/NTPs-driven transcription of RNA R_6_, which displaces P_5_ from the machinery T_5_, leading to negative feedback inhibition. The RNase H in the reaction circuit cleaves the R_6_/P_5_ (RNA/DNA) duplex, generating R_6_ fragments as “waste” and releasing P_5_ which acts as the promoter to reactivate transcription machinery T_5_. That is, the coupled operation of the two transcription machineries leads to the temporal oscillatory operation of the reaction module, while oscillatory modulating the formation and depletion of the template P_6_/T_6_, which includes the anti-thrombin aptamer inhibiting constituent. In parallel, the RNase H cleavage of the R_6_/P_5_ duplex leads to the reconfiguration of the transcription machinery P_5_/T_5_, completing the transient oscillatory features of the coupled transcription machineries. The oscillatory-modulated transient machineries are then assessed by extracting samples from the reaction mixture and probing their temporal activity/inhibition functions toward fibrinogen coagulation. This dynamic process is probed by following the temporal light-scattering changes associated with fibrinogenesis, providing insight into the oscillatory control of thrombin inhibition and fibrin formation.

Figure 5B shows the dynamic light-scattering intensity changes as a result of fibrinogen coagulation proceeding in samples withdrawn at time intervals from the oscillatory-modulated transcription circuits displayed in Figure 5A, following photoactivation (λ = 365 nm for 6 min). At time = 0, thrombin is active in catalyzing fibrinogen coagulation, as indicated by the fast light-scattering dynamic curve i. At time = 20 min, a slow light-scattering dynamic curve curve ii is observed, consistent with the inhibition of thrombin by the intermediate template P_6_/T_6_ in the system. Subsequently, at time = 100 min, the dynamic light-scattering rates are again enhanced, as seen in curve iv, demonstrating the reactivation of thrombin toward the coagulation of fibrinogen. Moreover, inspection of the different light-scattering dynamic curves reveals alternating slow/fast kinetic behaviors, consistent with an oscillatory pattern in the coagulation of fibrinogen by the system. (A control experiment displaying the time-dependent light-scattering changes in non-illuminated samples withdrawn at time intervals from the reaction mixture is presented in Figure S15.) Figure 5C,D presents the kinetic analysis of the light-scattering curves shown in Figure 5B, demonstrating the phototriggered temporal, oscillatory-modulated behaviors of the transcription machineries-guided coagulation of fibrinogen into fibrin. Figure 5C, curve I presents the *t*_50_ values (the time interval reaching the threshold of light-scattering intensity equal to 50 a.u.) corresponding to the dynamic light-scattering curves in Figure 5B. The system exhibits an oscillatory behavior with periodic increases and decreases in the light-scattering *t*_50_ values. (The analysis of the control system in the absence of photochemical deprotection is shown in curve II, demonstrating constant *t*_50_ values.) Figure 5D, curve I presents the temporal maximum coagulation rates, *V*_max_, of the light-scattering dynamic profiles shown in Figure 5B. An oscillatory behavior in the *V*_max_ values is observed, where a rapid initial decrease in *V*_max_ corresponds to the phototriggered transcription machineries-guided temporal inhibition of thrombin, followed by an oscillatory-modulated activation and inhibition process. Note that the rhythms of oscillations presented by *t*_50_ and *V*_max_ demonstrate opposite patterns. Moreover, the time gap between the first and second oscillating peaks (Δ*t*_1_) is approximately 3 h, whereas the time gaps separating the subsequent oscillating peaks (Δ*t*_*n*_, *n* ≥ 2) corresponds to ca. 2 h, which is consistent with the rhythms observed in the parent oscillatory transcription machineries.

### Probing the Phototriggered Oscillatory Transcription Circuits at Physiological-Like Conditions

The systems discussed so far were characterized in pure buffered aqueous solutions. The proposed application of phototriggered transient oscillatory transcription circuits for modulated control over thrombin-mediated coagulation of fibrinogen (fibrinogenesis) requires, however, probing the possible adaptation of these systems to operate under physiological-like conditions or in the presence of auxiliary perturbing environments existing in native systems. While the current systems are, certainly, far from immediate practical utility, we examined the feasibility of the phototriggered, oscillatory modulated systems to sustain such environmental perturbations.

**Figure 6:**
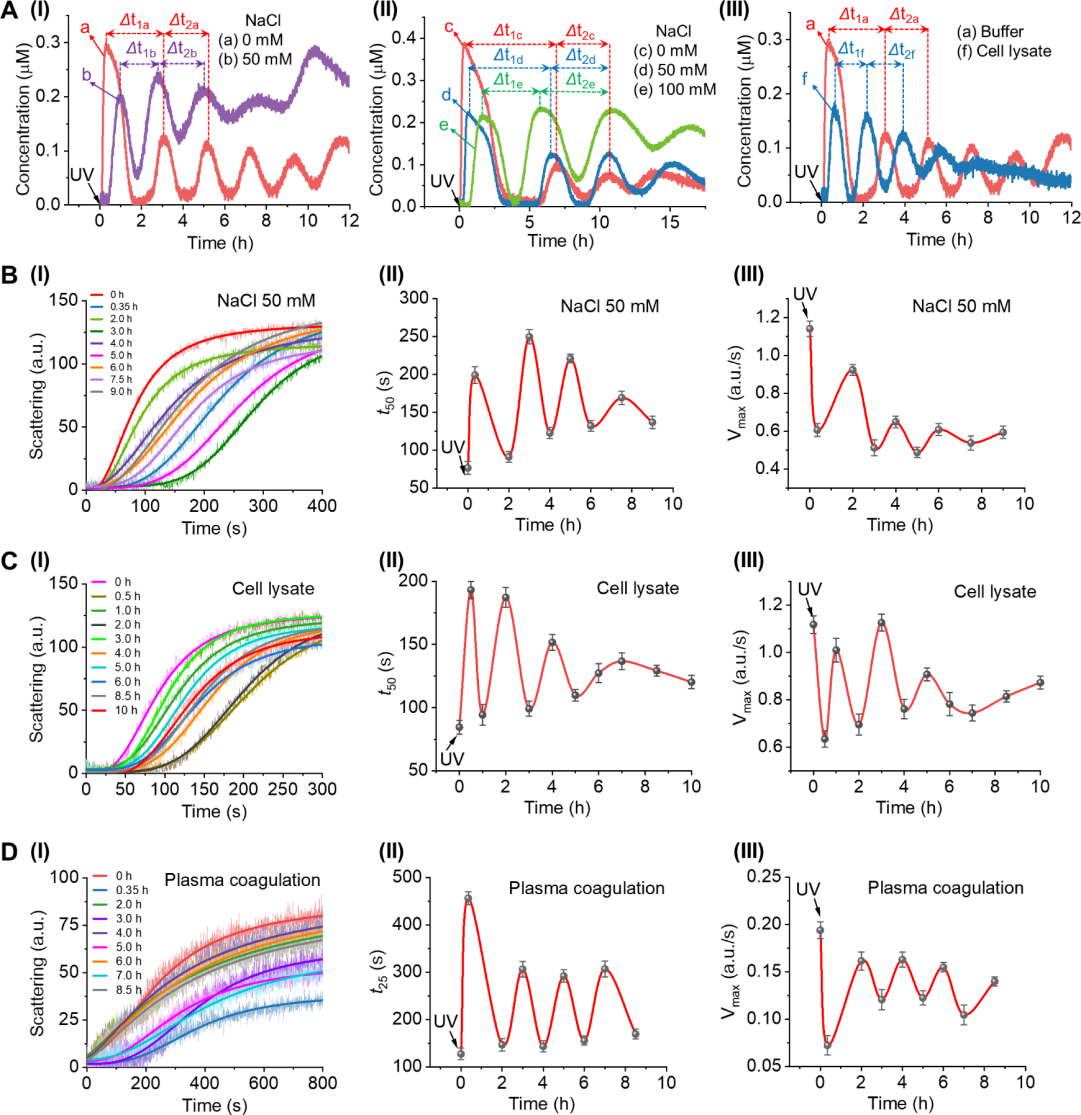
(A) Comparison of the performance of the phototriggered transient oscillatory transcription circuit displayed in Figure 3 under buffered conditions to its operation in the presence of environmental perturbances including: panels I and II—different salt concentrations, panel III—cell lysate. Operation conditions for panels I and III: T_3_* = 100 nM, P_3_ = 1.5 μM, B_4_ = 1.5 μM, P_4_ = 0.5 μM, T_4_ = 0.5 μM, T7 RNAP = 128 nM, RNase H = 1.00 nM, and NTPs = 7.5 mM at 35 °C, UV illumination (λ = 365 nm) for 6 min. Operation conditions for panel II: B_4_ = 1.0 μM, RNase H = 0.66 nM (other conditions are the same as the panel I). (B) and (C) Phototriggered transcription circuit-guided oscillatory modulated coagulation of fibrinogen to fibrin in the presence of (B) 50 mM NaCl, (C) cell lysate. (D) Phototriggered transcription circuit-guided oscillatory modulated coagulation of plasma. For (B)–(D), panels I depict the temporal light-scattering kinetic profiles corresponding to the fibrinogenesis processes in samples withdrawn from the reaction mixtures at different time intervals, panels II present the temporally oscillatory-modulated *t*_50_ or *t*_25_ values, and panels III display the temporally oscillatory-modulated *V*_max_ values.

Accordingly, we decided to evaluate the potential activation of the photochemically triggered transient oscillatory transcription machineries and the phototriggered, transcription circuit-modulated thrombin-mediated coagulation of fibrinogen to fibrin under physiological-like perturbing conditions, including elevated contents of salt and protein-containing cell lysates. Furthermore, the adaptivity of the phototriggered oscillatory transcription circuit-guided coagulation of fibrinogen to fibrin in human plasma samples was evaluated as a model for oscillatory modulated blood clotting in human fluid. The experimental results outlined below aim to demonstrate the feasibility of applying such systems for phototriggered temporal oscillatory fibrinogen coagulation, yet extensive optimization of the circuits will be needed. Figure 6A compares the performance of the phototriggered transcription machinery shown in Figure 3, operating in buffered aqueous solution and under perturbing conditions, including increased salt concentration (NaCl 50 mM, panels I and II) and in the presence of MDA-MB 231 breast cancer cell lysate (protein 1 mg/mL, panel III). Evidently, the oscillation patterns in the presence of NaCl 50 mM (panel I) follow those in the buffered solution, though the amplitude of the modulated output is dampened and the rhythm of oscillation is slightly shifted. Moreover, panel II demonstrates that further increases in NaCl concentration retain the rhythm of oscillations but affect the appearance time of the first modulated peaks and the time gaps separating the subsequent oscillating peaks. Panel III compares the performance of the phototriggered oscillatory transcription circuit in the buffered solution and in the presence of cell lysate. The oscillatory behavior in cell lysate indicates the successful operation of the phototriggered oscillatory transcription circuit in the cell lysate medium. Nevertheless, the cell lysate has an effect on the amplitudes of the modulated transcription circuit and causes a temporal amplitude shift compared to the buffered solution. Obviously, the results indicate that the phototriggered oscillatory transcription circuit retains to function under physiological-like conditions, although the environmental parameters affect the dynamics of the circuit. At present, the origin of these oscillatory perturbations is unknown. Presumably, these conditions affect the catalytic properties of the enzymes or the duplex stabilities of the constituents involved in the transcription framework. Future characterization of these perturbations on the oscillatory modulated process, and eventually integration of these effects into the kinetic models associated with the oscillatory system could lead to an optimized oscillatory circuit operating effectively under auxiliary perturbations. Figure 6B–D displays the phototriggered transcription circuit-guided oscillatory thrombin-catalyzed coagulation of fibrinogen to fibrin, according to Figure 5A under buffered conditions, operating under high salt concentration, Figure 6B, in cell lysate media, Figure 6C, and even applying the circuit for coagulation of human plasma, Figure 6D. In general, the phototriggered, transcription circuit-guided, oscillatory modulated coagulation of fibrinogen under auxiliary perturbing conditions follows the oscillatory modulated process observed under buffered conditions as presented in Figure 5C. However, minor effects are observed, including differences in the amplitudes of the modulated peaks and varying time gaps between the oscillating peaks. Most importantly, the oscillatory modulated coagulation of fibrinogen in human plasma environment, Figure 6D, almost fully overlaps with that in buffered conditions, suggesting the future feasibility of applying the system for therapeutic uses. (For additional experiments comparing the performance of the phototriggered, transient, non-oscillatory, transcription machinery-driven coagulation of fibrinogen, Figure 2, with the performance of the system under environmental perturbances, see Figure S16. Furthermore, the effects of HSA on the phototriggered oscillatory transcription machinery and the phototriggered transcription circuit-guided oscillatory modulated coagulation of fibrinogen are provided in Figure S17.

## Conclusions

The present study has introduced photochemically triggered transient and oscillatory temporally modulated transcription machineries driven by synthetic DNA reaction modules and circuits. Besides modeling native transcription machineries by demonstrating dynamically adaptive, modulated RNA expression pathways driven by auxiliary triggers, the study advanced the topic of dynamic DNA machineries by introducing the following innovative elements: (i) it introduced caged DNA hairpin structures enabling the phototriggered emergence of functional transcription templates, allowing the transient operation or temporally oscillatory-modulated transient machineries synthesizing predesigned RNA products. Within the extensive efforts to develop dynamic and transient transcription machineries, oscillatory modulated transient transcription machineries are unprecedented. Moreover, our results demonstrate the capacity to operate these dynamic processes in physiological-like perturbing environments. Accordingly, these concepts may be adopted for the spatiotemporal activation of transcription machineries in cell compartments and target tissues. The transient and temporally oscillatory-modulated transcription circuits were computationally simulated by kinetic models. The computational simulations not only provided kinetic parameters associated with the complex dynamic circuits but also allowed the prediction and subsequent experimental validation of the reaction circuits under different auxiliary conditions. (ii) The dynamic transcription circuits were applied as machineries controlling the transient thrombin-mediated or temporally oscillatory-modulated thrombin-induced fibrinogenesis. These results spark potential therapeutic applications of the dynamic transcription machineries by controlling the temporal and dose-controlled synthesis of intermediate RNA products that modulate fibrinogenesis for wound healing and blood clotting. Moreover, the transient and temporally modulated transcription of an RNA product might be further translated into DNAzyme catalytic units,^27,65^ thereby providing new temporal or oscillatory catalytic tools.

Beyond these advances, important challenges are still ahead of us. The coupling of the transcription machineries to transient or temporally oscillatory-modulated translation of proteins is an interesting path to follow for developing spatiotemporal therapies. In addition, substantial recent efforts are directed toward developing synthetic cell analogs (protocells). Diverse synthetic carriers, such as liposomes,^66,67^ polymersomes,^68,69^ dendrosomes,^70^ microcapsules,^26^ proteinsomes,^71,72^ microdroplets,^36^ and condensates,^73,74^ were introduced as biomimetic cell constructs. The integration of dynamic transcription circuits into such protocells is an interesting path to explore. Particularly, recent advancements addressed the fusion of partially loaded protocells and the emergence of complex circuit-loaded containments^75^ or the fusion of loaded liposomes with cells and delivery of the liposomes loads into the cells.^76^ The emergence of synthetic transcription circuits in such containments and their delivery into native cells are anticipated to introduce diverse therapeutic applications.

## Supporting Information Available

Experimental section; DNA sequences; detailed preparations of the light-triggered transient and oscillatory transcription reaction mixtures; methods for the light-triggered, transcription-guided transient and oscillatory fibrogenesis; gel electrophoresis; calibration curves; computational kinetic models and the sets of derived rate constants; control experiments and complementary explanatory results (PDF)
