# Supplementary material for: Photochemically Triggered, Transient, and Oscillatory Transcription Machineries Guide Temporal Modulation of Fibrinogenesis
Source: J Am Chem Soc. 2024 Dec 31;147(2):2216–27. doi: 10.1021/jacs.4c16829 (PMC11744759; doi:10.1021/jacs.4c16829)
Supplement: Supplementary file 1 — ja4c16829_si_001.pdf [file ja4c16829_si_001.pdf]

Supporting Information

Photochemically Triggered, Transient, and Oscillatory Transcription Machineries Guide Temporal Modulation of Fibrinogenesis

Jiantong Dong and Itamar Willner*

Institute of Chemistry, The Hebrew University of Jerusalem, Jerusalem 91904, Israel

*Email: itamar.willner@mail.huji.ac.il

Materials and Methods

Chemicals

T7 RNA polymerase (T7 RNAP, 50,000 U/mL, 800 nM), RNase H (5,000 U/mL, 166 nM), ribonucleotide (NTP) Mix (GTP, ATP, UTP and CTP, each 25 mM), and 10 × RNAPol reaction buffer (400 mM Tris-HCl, 60 mM $MgCl_2$, 10 mM DTT, 20 mM spermidine, pH 7.9 @ 25 °C) were purchased from New England BioLabs Inc. Thrombin from human plasma (≥ 2,000 NIH units/mg protein, MW = 37.4 kDa), fibrinogen from human plasma (50–70% protein), acrylamide/bis-acrylamide (40% solution, 19:1), ammonium persulfate, *N,N,N′,N′*-tetramethylethylenediamine, magnesium chloride ($MgCl_2$), calcium chloride ($CaCl_2$), albumin from human serum (HSA), and human plasma powder were purchased from Sigma-Aldrich. DNA oligonucleotides used in the study were custom ordered from Integrated DNA Technologies Inc. MDA-MB-231 breast cancer cell lysates (containing 1 mg/mL protein) were prepared by sonicating the cells in a buffer solution (100 mM NaCl, 40 mM Tris-HCl, 6 mM $MgCl_2$, 1 mM DTT, pH 7.9), followed by filtration to remove the cell membrane.

Instrumentation

The concentrations of DNA were measured using NanoDrop™ 2000/2000c Spectrophotometers (Thermo Scientific™). UV light source was carried on THORLABS device including LED (SM1U25-A) and driver (M01018702) with output 20 mW/cm^2. Fluorescence spectra, kinetics, and light-scattering intensity were recorded using a Cary Eclipse Fluorometer (Varian Inc). Quartz cuvettes (Ultra-Micro Fluorescence Cell, Hellma Analytics, Germany) and plastic cuvettes (disposable cuvette, Brand GMBH, Wehrheim, Germany) were used for the fluorescence and light-scattering measurement, respectively. Gel electrophresis were performed using omniPAGE Mini Vertical Electrophoresis System and imaged using a Fusion FX-Vilber Lumart instrument.

Summary of nucleic acid strands used in this study

T_1*	5′-GACAGAAGAACGCTGAGGCCATCCCTATAGTG/**iSpPC**/AGTATC*TAATACG*ACTCACTATAGGGATGGCCTCAGCGTTCTTCTGTC-3′
P_1	5′-AGTCGTATTAGATAG-3′

L_1	5′-Cy3-GACAGAAGAACGCTGAGGCCATC-3′
L_1′	5′-GATGGCCTCAGCGTT-BHQ2-3′
T_1-t	5′-GACAGAAGAACGCTGAGGCCATCCCTATAGTG-3′
T_1-nt	5′-AGTATC*TAATACG*ACTCACTATAGGGATGGCCTCAGCGTTCTTCTGTC-3′
T_2*	5′-GGTTGGTGTGGTTGGCCCTATAGTG/**iSpPC**/AGTATC*TAATACG*ACTCACTATAGGGCCAACCACACCAACC-3′
A_2	5′-GGTTGGTGTGGTTGG-3′
T_3*	5′-GTGTGTAGTAGTAGTTTCGTTCACCCTATAGTGAGTCG/**iSpPC**/GAAGGAAAGA*TAATA*CGACTCACTATAGGGTGAACGAAACTACTACTACACA-3′
P_3	5′-TATTATCTTTCCTTCCAATCCGT-3′
T_4-t	5′-TATTATCTTTCCTTCCAATCCGTCCCTATAGTGAGTCG-3′
T_4-nt	5′-TexasRed-TCGTTCACAGTAATACGACTCACTATAGGGACGGATTGGAAGGAAAGATAATA-3′
P_4	5′-TATTACTGTGAACGAAACTACTAC-Iowa Black®RQ-3′
B_4	5′-GTGTGTAGTAGTAGTTTCGTTCACAGTA-3′
T_5*	5′-GTCTCTTACCCAACCTAGGCGTGACTCCCTATAGTGAGTCG/**iSpPC**/GAAGGAAAGA*TAATA*CGACTCACTATAGGGAGTCACGCCTAGGTTGGGTAAGAGAC-3′
P_5	5′-TATTATCTTTCCTTCCAATCCGT-3′
T_6-t	5′-TATTATCTTTCCTTCCAATCCGTCCCTATAGTGAGTCG-3′
T_6-nt	5′-AGGGTGGTGGCGTGACTCTAATACGACTCACTATAGGGACGGATTGGAAGGAAAGATAATA-3′
P_6	5′-TATTAGAGTCACGCCTAGGTTGGGT-3′
B_6	5′-GTCTCTTACCCAACCTAGGCG TGACTCTA-3′
Note	The photoresponsive chemical modification is shown in bold. The promoter sequences are marked in green color, and the sequence caged in loop of hairpin template is presented in italics. The subunits of the anti-thrombin aptamer are marked in orange color.

Methods

Preparation and measurement of photochemically triggered transient transcription machinery

T_1* (20 μM) in 1× RNAPol reaction buffer was annealed at 90 °C for 5 min and then cooled down to 25 °C over 30 min. L_1/L_1′ (40 μM) was annealed following the same procedure. A mixture solution (50 μL) consisting of T_1* (0.4 μM), P_1 (0.4 μM), T7 RNAP (96 nM, 6 U/μL), and RNase H (0.40 nM, 12 U/mL) in 1× RNAPol reaction buffer (20 mM $MgCl_2$) was illuminated under UV light (λ = 365 nm, 20 mW/cm^2) for different time intervals. It should be noted that the low-power light source used to uncage the *ortho*-nitrobenzyl phosphate ester-modified strand did not lead to any electrophoretically detectable side

products of the nucleic acid strands. Afterward, the photo-activated reaction mixture was subjected to a solution (50 μL) containing L_1/L_1' (3.2 μM) and NTPs (1 mM) in 1× RNAPol reaction buffer (20 mM $MgCl_2$). The final photo-triggered transient transcription system (100 μL) is composed of T_1 (0.2 μM), P_1 (0.2 μM), T7 RNAP (48 nM), RNase H (0.20 nM), L_1/L_1' (1.6 μM), and NTPs (0.5 mM). Time-dependent fluorescence changes (λ_{ex} 540 nm/λ_{em} 562 nm) were immediately monitored on a Cary Eclipse Fluorometer (voltage 700 V) at 35 °C using quartz cuvettes with 10-mm path lengths. The temporal concentrations of the displaced L_1 were quantified by using an appropriate calibration curve corresponding to the fluorescence changes of Cy3-labeled L_1 (1.6 μM) upon adding different concentrations (0–1.6 μM) of BHQ2-labeled L_1'.

Preparation and assessment of photochemically triggered, transcription machinery-guided transient fibrinogenesis

T_2* (20 μM) in 1× RNAPol reaction buffer was annealed at 90 °C for 5 min, then cooled down to 25 °C over 30 min. A reaction mixture solution (500 μL) consisting of T_2* (0.2 μM), P_1 (0.2 μM), A_2 (0.5 μM), thrombin (10 nM), T7 RNAP (32 nM, 2 U/μL), RNase H (0.20 nM), and NTPs (0.5 mM) in 1× RNAPol reaction buffer (20 mM $MgCl_2$) was illuminated under UV light (λ = 365 nm, 20 mW/cm^2) for different time intervals, then incubated at 35 °C. To probe the fibrinogenesis regulated by the photo-activated dissipative transcription machinery, 50 μL aliquots of the reaction mixture were withdrawn at time intervals and mixed with 50 μL of fibrinogen (1 mg/mL) in 1× RNAPol reaction buffer (20 mM $MgCl_2$) in disposable plastic cuvettes. The clotting reaction was immediately monitored by measuring the changes of scattered light intensities at 650 nm using a Cary Eclipse Fluorometer (voltage 500 V) at 25 °C.

To study the effect of NaCl (50 mM) on the photochemically triggered, transcription machinery-guided transient fibrinogenesis, a reaction mixture solution (300 μL) consisting of T_2* (0.2 μM), P_1 (0.2 μM), A_2 (0.25 μM), thrombin (10 nM), T7 RNAP (48 nM, 3 U/μL), RNase H (0.20 nM, 6U/mL), and NTPs (0.5 mM) in 1× RNAPol reaction buffer (20 mM $MgCl_2$) with NaCl (50 mM) was illuminated under UV light (λ = 365 nm, 20 mW/cm^2) for 6 min, then incubated at 35 °C. To probe the fibrinogenesis, 50 μL aliquots of the reaction

mixture were withdrawn at time intervals and mixed with 50 μL of fibrinogen (1 mg/mL) in 1× RNAPol reaction buffer (20 mM $MgCl_2$) with NaCl (50 mM) in disposable plastic cuvettes. The clotting reaction was immediately monitored by measuring scattered light intensities at 650 nm using a Cary Eclipse Fluorometer (voltage 500 V) at 25 °C.

To study the effect of HSA (1 mg/mL) on the photochemically triggered, transcription machinery-guided transient fibrinogenesis, a reaction mixture solution (300 μL) consisting of T_2* (0.2 μM), P_1 (0.2 μM), A_2 (0.5 μM), thrombin (10 nM), T7 RNAP (48 nM, 3 U/μL), RNase H (0.20 nM, 6U/mL), and NTPs (0.5 mM) in 1× RNAPol reaction buffer (20 mM $MgCl_2$) with HSA (1 mg/mL) was illuminated under UV light (λ = 365 nm, 20 mW/cm^2) for 6 min, then incubated at 35 °C. To probe the fibrinogenesis, 50 μL aliquots of the reaction mixture were withdrawn at time intervals and mixed with 50 μL of fibrinogen (1 mg/mL) in 1× RNAPol reaction buffer (20 mM $MgCl_2$) in disposable plastic cuvettes. The clotting reaction was immediately monitored by measuring scattered light intensities at 650 nm using a Cary Eclipse Fluorometer (voltage 500 V) at 25 °C.

To study the effect of cell lysate (containing 1 mg/mL protein, suspended in a buffer composed of 100 mM NaCl, 40 mM Tris-HCl, 6 mM $MgCl_2$, 1 mM DTT, pH 7.9) on the photochemically triggered, transcription machinery-guided transient fibrinogenesis, a reaction mixture solution (300 μL) consisting of T_2* (0.2 μM), P_1 (0.2 μM), A_2 (0.25 μM), thrombin (10 nM), T7 RNAP (48 nM, 3 U/μL), RNase H (0.20 nM, 6 U/mL), and NTPs (0.5 mM) in 1× RNAPol reaction buffer (20 mM $MgCl_2$) with 150 μL cell lysate was illuminated under UV light (λ = 365 nm, 20 mW/cm^2) for 6 min, then incubated at 35 °C. To probe the fibrinogenesis, 50 μL aliquots of the reaction mixture were withdrawn at time intervals and mixed with 50 μL of fibrinogen (1 mg/mL) in 1× RNAPol reaction buffer (20 mM $MgCl_2$) in disposable plastic cuvettes. The clotting reaction was immediately monitored by measuring scattered light intensities at 650 nm using a Cary Eclipse Fluorometer (voltage 500 V) at 25 °C.

To apply the photo-triggered transient system for coagulation of human plasma, a reaction mixture solution (400 μL) consisting of T_2* (0.2 μM), P_1 (0.2 μM), A_2 (0.5 μM), thrombin (10 nM), T7 RNAP (32 nM, 2 U/μL), RNase H (0.20 nM), and NTPs (0.5 mM) in

1× RNAPol reaction buffer (20 mM $MgCl_2$) was illuminated under UV light (λ = 365 nm, 20 mW/cm^2) for 6 min, then incubated at 35 °C. To probe the coagulation of human plasma, 50 μL aliquots of the reaction mixture were withdrawn at time intervals and mixed with 50 μL of human plasma solution (supplied with 30 mM $CaCl_2$ to neutralize the 4% trisodium citrate) in disposable plastic cuvettes. The clotting reaction was immediately monitored by measuring the changes of scattered light intensities at 650 nm using a Cary Eclipse Fluorometer (voltage 450 V) at 25 °C.

Preparation and monitoring of photo-activated oscillatory transcription circuit

T_3* (10 μM) in 1 × RNAPol reaction buffer was annealed at 90 °C for 5 min, then cooled down to 25 °C over 30 min. T_4 (20 μM) consisting of $T_{4\text{-nt}}$ and $T_{4\text{-t}}$ followed the same annealing procedure. A mixture solution (60 μL) consisting of T_3* (0.14 μM), P_3 (3 μM), T7 RNAP (256 nM, 16 U/μL), and RNase H (2.00 nM, 60 U/mL) in 1× RNAPol reaction buffer (20 mM $MgCl_2$) was illuminated under UV light (λ = 365 nm, 20 mW/cm^2) for different time intervals. Afterward, the photo-activated reaction mixture was subjected to a solution (60 μL) containing T_4 (1 μM), P_4 (1 μM), B_4 (3 μM), and NTPs (15 mM) in 1× RNAPol reaction buffer (20 mM $MgCl_2$). The final photo-activated oscillatory transcription circuit system (120 μL) is composed of T_3 (0.07 μM), P_3 (1.5 μM), B_4 (1.5 μM), P_4 (0.5 μM), T_4 (0.5 μM), T7 RNAP (128 nM), RNase H (1.00 nM), and NTPs (7.5 mM). Time-dependent fluorescence changes (λ_{ex} 594 nm/λ_{em} 614 nm) were immediately monitored on a Cary Eclipse Fluorometer (voltage 700 V) at 35 °C using quartz cuvettes with 10-mm path lengths. The temporal concentrations of the intermediate transcription template P_4/T_4 were quantified by using an appropriate calibration curve corresponding to the fluorescence changes of TexasRed-labeled T_4 (0.5 μM) upon adding variable concentrations (0–0.5 μM) of Iowa Black®RQ-labeled P_4.

Preparation and assessment of photo-triggered, transcription circuit-guided, oscillatory modulated fibrinogenesis

T_5* (10 μM) in 1 × RNAPol reaction buffer was annealed at 90 °C for 5 min, then cooled down to 25 °C over 30 min. T_6 (20 μM) consisting of $T_{6\text{-nt}}$ and $T_{6\text{-t}}$ followed the same annealing procedure. A reaction mixture solution (400 μL) consisting of T_5* (0.15 μM), P_5

(1.5 μM), B_6 (1.5 μM), P_6 (0.5 μM), T_6 (0.5 μM), T7 RNAP (128 nM), RNase H (1.00 nM), and NTPs (7.5 mM) in 1× RNAPol reaction buffer (20 mM $MgCl_2$) was illuminated under UV light (λ = 365 nm, 20 mW/cm^2) for 6 min, then incubated at 35 °C. To probe the fibrinogenesis regulated by the photo-triggered oscillatory transcription circuit, 30 μL aliquots of the reaction mixture were withdrawn at time intervals and incubated with 64 μL of 1× RNAPol reaction buffer (20 mM $MgCl_2$) and 1 μL of thrombin (0.5 μM) for 10 min. After that, 5 μL of fibrinogen (10 mg/mL) were added to the mixture. The clotting reaction was immediately monitored by measuring the changes of scattered light intensities at 650 nm using a Cary Eclipse Fluorometer (voltage 500 V) at 25 °C.

To study the effect of NaCl (50 mM) or HSA (1 mg/mL) on the photochemically triggered, transcription circuit-guided, oscillatory modulated fibrinogenesis, a mixture solution (250 μL) consisting of T_5* (0.10 μM), P_5 (1.5 μM), B_6 (1.5 μM), P_6 (0.5 μM), T_6 (0.5 μM), T7 RNAP (128 nM), RNase H (1.00 nM), and NTPs (7.5 mM) in 1× RNAPol reaction buffer (20 mM $MgCl_2$) with NaCl (50 mM) or HSA (1 mg/mL) was illuminated under UV light (λ = 365 nm, 20 mW/cm^2) for 6 min, then incubated at 35 °C. To probe the fibrinogenesis, 25 μL aliquots of the reaction mixture were withdrawn at time intervals and incubated with 69 μL of 1× RNAPol reaction buffer and 1 μL of thrombin (0.5 μM) for 10 min. After that, 5 μL of fibrinogen (10 mg/mL) were added to the mixture in a disposable plastic cuvette. The clotting reaction was immediately monitored by measuring the changes of scattered light intensities at 650 nm using a Cary Eclipse Fluorometer (voltage 480 V for NaCl 50 mM, 500 V for HSA 1 mg/mL) at 25 °C.

To study the effect of cell lysate (containing 1 mg/mL protein, suspended in a buffer composed of 100 mM NaCl, 40 mM Tris-HCl, 6 mM $MgCl_2$, 1 mM DTT, pH 7.9) on the photochemically triggered, transcription circuit-guided, oscillatory modulated fibrinogenesis, a mixture solution (300 μL) consisting of T_5* (0.10 μM), P_5 (1.5 μM), B_6 (1.5 μM), P_6 (0.5 μM), T_6 (0.5 μM), T7 RNAP (128 nM), RNase H (1.00 nM), and NTPs (7.5 mM) in 1× RNAPol reaction buffer (20 mM $MgCl_2$) with 100 μL cell lysate was illuminated under UV light (λ = 365 nm, 20 mW/cm^2) for 6 min, then incubated at 35 °C. To probe the fibrinogenesis, 25 μL aliquots of the reaction mixture were withdrawn at time

intervals and incubated with 69 μL of 1× RNAPol reaction buffer and 1 μL of thrombin (0.5 μM) for 10 min. After that, 5 μL of fibrinogen (10 mg/mL) were added to the mixture. The clotting reaction was immediately monitored by measuring the scattered light intensities at 650 nm using a Cary Eclipse Fluorometer (voltage 500 V) at 25 °C.

To apply the oscillatory system for coagulation of human plasma, a reaction mixture solution (200 μL) consisting of T_2* (0.2 μM), P_1 (0.2 μM), A_2 (0.5 μM), thrombin (10 nM), T7 RNAP (32 nM, 2 U/μL), RNase H (0.20 nM), and NTPs (0.5 mM) in 1× RNAPol reaction buffer (20 mM $MgCl_2$) was illuminated under UV light (λ = 365 nm, 20 mW/cm^2) for 6 min, then incubated at 35 °C. To probe the coagulation of human plasma, 20 μL aliquots of the reaction mixture were withdrawn at time intervals and incubated with 40 μL of 1× RNAPol reaction buffer and 1 μL of thrombin (0.5 μM) for 10 min. After that, 40 μL of human plasma solution (supplied with 35 mM $CaCl_2$ to neutralize the 4% trisodium citrate) was added to the mixture in a disposable plastic cuvette. The clotting reaction was immediately monitored by measuring the changes of scattered light intensities at 650 nm using a Cary Eclipse Fluorometer (voltage 480 V) at 25 °C.

Gel electrophoresis for UV-uncaging of the PC-modified hairpin template

Aliquots of 10 μL reaction mixture were prepared and mixed with 1× Gel loading buffer. Native polyacrylamide gel electrophoresis experiments were performed using polyacrylamide gel (12%, 19:1 acrylamide/bis-acrylamide) and run using an omniPAGE Mini Vertical Electrophoresis System at 80 V and at 5 °C for 2–5 h. Following electrophoresis, DNA strands were visualized by staining with 1 × GelRed and imaged using a Fusion FX-Vilber Lumart instrument.

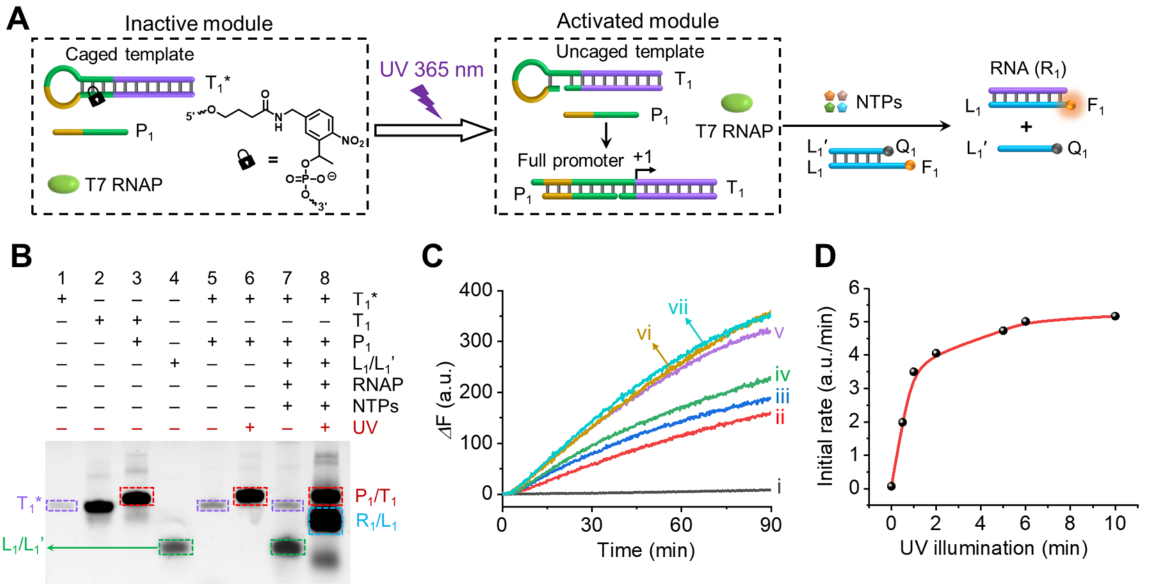

Figure S1. (A) Panel I: Schematic of the photochemical uncaging of the transcription template T_1* and the activation of the transcription machinery. (B) Gel electrophoresis separation of the reaction module shown in (A) and the control experiments: Lane 1 – T_1* (0.5 μM); Lane 2 – T_1 (1.0 μM); Lane 3 – P_1/T_1 (1.0 μM); Lane 4 – L_1/L_1′ (1.0 μM); Lane 5 – T_1* (1.0 μM) and P_1 (1.0 μM) without UV illumination; Lane 6 – T_1* (1.0 μM) and P_1 (1.0 μM) with UV illumination (λ = 365 nm) for 10 min; Lane 7 – inactive reaction module incubated at 35 °C for 2 h without UV illumination; Lane 8 – photo-activated transcription module incubated at 35 °C for 2 h after UV illumination (λ = 365 nm) for 10 min. Experimental conditions for Lanes 7 and 8: T_1* = 1.0 μM, P_1 = 1.0 μM, L_1/L_1′ = 2.0 μM, T7 RNAP = 32 nM, NTPs = 0.5 mM. (C) Time-dependent fluorescence changes generated by the displacement of Cy3-labeled L_1′ by the transcription product R_1 after UV illumination for different time intervals: (i) 0, (ii) 0.5, (iii) 1, (iv) 2, (v) 5, (vi) 6, and (vii) 10 min. (D) The initial transcription rates (within a period of 5–10 min) corresponding to the curves shown in (C) following different UV illumination time intervals.

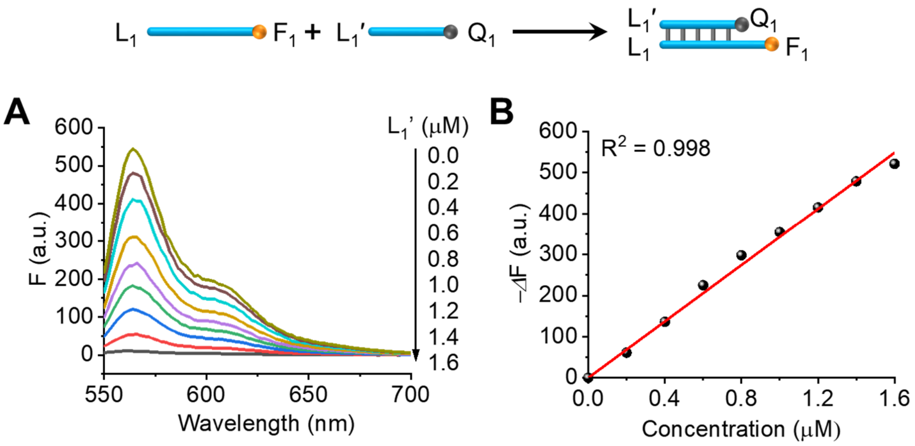

Figure S2. (A) The fluorescence spectra of Cy3-labeled L_1 (1.6 μM) upon the addition of variable concentrations of BHQ2-labeled L_1', leading to a decrease in the fluorescence intensity (λ_{ex} = 540 nm). (B) The derived calibration curve relating the decrease in fluorescence intensity (λ_{em} = 562 nm) in the presence of variable concentrations of BHQ2-labeled L_1'.

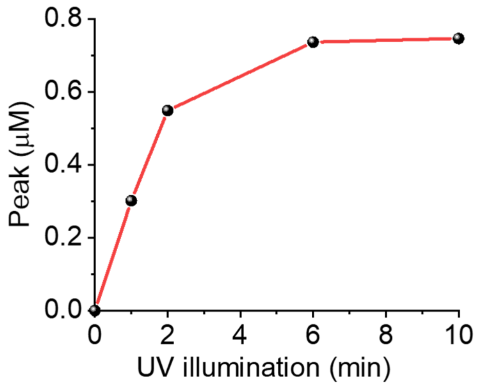

Figure S3. Peak concentrations of the intermediate product R_1/L_1 generated by UV illumination of the reaction module for different time intervals, according to Figure 1B.

Kinetic simulation of experimental results

Kinetic models for the photochemically triggered, transient, and oscillatory-modulated transcription machineries were formulated. The initial constituent concentrations for simulation of each system are detailed for each formulated model. The simulations are based on the law of mass conservation of intermediates.

(i) Rate constants for each system were determined by an optimized fitting curve overlaid on the experimental result.

(ii) To ensure the derived set of rate constants was valid and not just coincidental, we used the set of rate constants to predict the system's behavior under different auxiliary conditions and validated these predictions experimentally.

(iii) We further support the set of rate constants by experimentally characterizing the sub-reactions that could be performed independently. Specifically, for the kinetic model of photochemically triggered transient transcription machinery described in Figure S4, the sub-reaction (2) was experimentally validated. The reaction module validating the constant k_2 was described in Figure S5, where the experimental rate constant is same as the computationally derived rate constant. (Details of this experiment are provided in Figure S5.) Additionally, the transcription reaction (without RNase H) was performed to validate the rate constants k_3, k_4, k_{-4}, and k_5. (Details of this experiment are provided in Figure S6).

(iv) Since the photochemically triggered transient transcription machinery and oscillatory-modulated transcription circuit share several common rate constants but require additional sub-reactions to address the complexity of oscillatory transcription circuit, these common rate constants were sequentially optimized across all systems. This ensured a global set of consistent rate constants applicable to all systems studied. The sets of rate constants for each system are provided in Tables S1 and S2, respectively.

Kinetic equations of photochemically triggered transient transcription machinery:

(1) $T_1 + P_1 \xrightarrow{k_1} P_1T_1$

(2) $L_1 + L_1' \xrightarrow{k_2} L_1L_1'$

(3) $R_1 + L_1L_1' \xrightarrow{k_3} R_1L_1 + L_1'$

(4) $P_1T_1 + \text{RNAP} \underset{k_{-4}}{\overset{k_4}{\rightleftharpoons}} P_1T_1\text{•RNAP}$

(5) $P_1T_1\text{•RNAP} + \text{NTPs} \xrightarrow{k_5} \text{RNAP} + P_1T_1 + R_1$ (N_{max} (G) = 8, yield (Y) =7%)

(6) $R_1L_1 + \text{RNaseH} \underset{k_{-6}}{\overset{k_6}{\rightleftharpoons}} R_1L_1\text{•RNaseH}$

(7) $R_1L_1\text{•RNaseH} \xrightarrow{k_7} \text{RNaseH} + L_1$

Derivatives:

$$\frac{dT_1}{dt} = -k_1[T_1][P_1]$$

$$\frac{dP_1}{dt} = -k_1[T_1][P_1]$$

$$\frac{dP_1T_1}{dt} = k_1[T_1][P_1] - k_4[P_1T_1][\text{RNAP}] + k_{-4}[P_1T_1\text{•RNAP}] + k_5[P_1T_1\text{•RNAP}][\text{NTPs}]/N_{max} \times Y$$

$$\frac{dL_1}{dt} = -k_2[L_1][L_1'] + k_7[R_1L_1\text{•RNaseH}]$$

$$\frac{dL_1'}{dt} = -k_2[L_1][L_1'] + k_3[R_1][L_1L_1']$$

$$\frac{dL_1L_1'}{dt} = k_2[L_1][L_1'] - k_3[R_1][L_1L_1']$$

$$\frac{dR_1}{dt} = -k_3[R_1][L_1L_1'] + k_5[P_1T_1\text{•RNAP}][\text{NTPs}] \times \text{Y}$$

$$\frac{dR_1L_1}{dt} = k_3[R_1][L_1L_1'] - k_6[R_1L_1][\text{RNaseH}] + k_{-6}[R_1L_1\text{•RNaseH}]$$

$$\frac{d\text{RNAP}}{dt} = k_{-4}[P_1T_1\text{•RNAP}] - k_4[P_1T_1][\text{RNAP}] + k_5[P_1T_1\text{•RNAP}][\text{NTPs}]/N_{max} \times Y$$

$$\frac{dP_1T_1\text{•RNAP}}{dt} = k_4[P_1T_1][\text{RNAP}] - k_{-4}[P_1T_1\text{•RNAP}] - k_5[P_1T_1\text{•RNAP}][\text{NTPs}]/N_{max} \times Y$$

$$\frac{d\text{NTPs}}{dt} = -k_5[P_1T_1\text{•RNAP}][\text{NTPs}]$$

$$\frac{d\text{RNaseH}}{dt} = k_{-6}[R_1L_1\text{•RNaseH}] - k_6[R_1L_1][\text{RNaseH}] + k_7[R_1L_1\text{•RNaseH}]$$

$$\frac{dR_1L_1\text{•RNaseH}}{dt} = k_6[R_1L_1][\text{RNaseH}] - k_{-6}[R_1L_1\text{•RNaseH}] - k_7[R_1L_1\text{•RNaseH}]$$

Figure S4. Computational simulation of the photochemically triggered transient transcription machinery. The kinetic scheme of the sub-reactions associated with the time-dependent concentration changes are summarized in the above equations. Knowing the time-dependent concentration changes of the R_1/L_1 during transcription, we computationally simulated the system by using Matlab R2019b. Initial concentrations of the condition: $T_1^* = T_1 = 0.2$ µM, $P_1 = 0.2$ µM, $L_1/L_1' = 1.6$ µM, T7 RNAP = 48 nM, RNase H = 0.20 nM, NTPs = 0.5 mM. The derived rate constants are summarized in Table S1. According to our previously study,[1] the RNA yield (*Y*), *i.e.*, the conversion efficiency of NTPs into RNA, was calculated as 7%.

Experimental validation of the rate constants k_2

To experimentally validate the rate constant k_2 appearing in the kinetic model of Figure S4, we subjected the Cy3-labeled L_1 (0.5 μM) to two concentrations of BHQ2-labeled L_1' (0.2 μM and 0.4 μM). The time-dependent concentration changes of L_1/L_1' were monitored by following the time-dependent fluorescence changes of Cy3 (λ_{em} = 562 nm) using the appropriate calibration curve in Figure S5B. Using the Matlab R2019b program, the rate constant k_2 = 26.0 μM^{-1} min^{-1} was derived. The experimental result well supports the computationally simulated rate constant k_2 = 26.0 μM^{-1} min^{-1} summarized in Table S1.

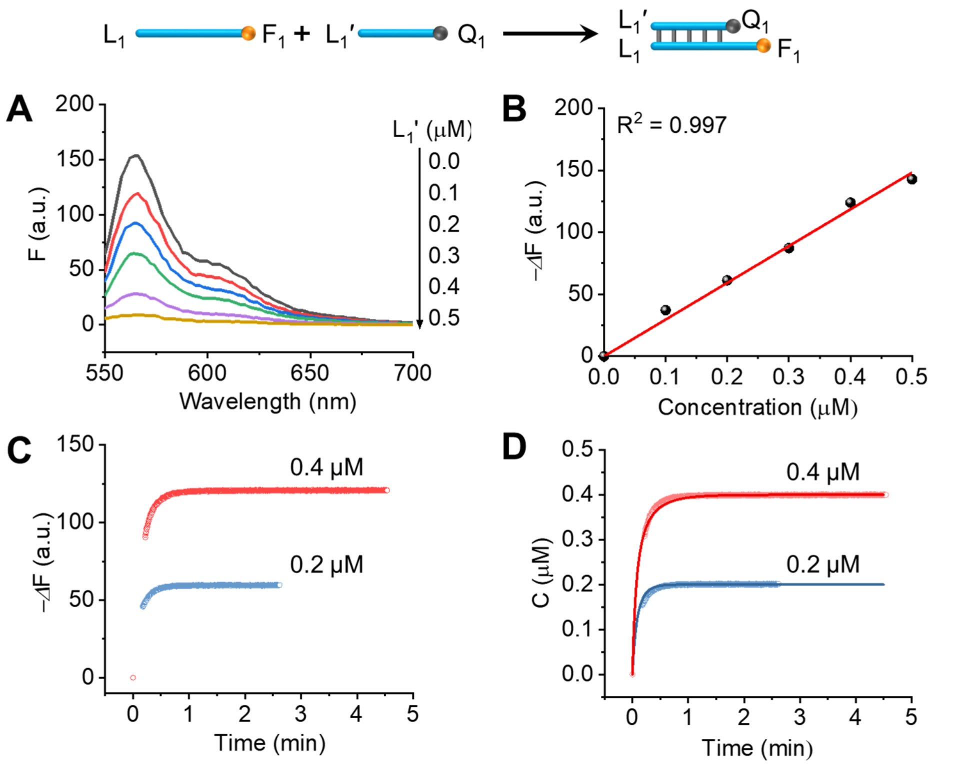

Figure S5. (A) The fluorescence spectra of Cy3-labeled L_1 (0.5 μM) upon binding to variable concentrations of BHQ2-labeled L_1'. (B) The derived calibration curve corresponding to the decrease in fluorescence intensity (λ_{em} = 562 nm) at different concentrations of L_1'. (C) Time-dependent fluorescence changes of L_1 and (D) time-dependent concentration changes of L_1/L_1' when L_1 (0.5 μM) is subjecting to L_1': 0.2 μM (blue) and 0.4 μM (red). Dots represent experimental data, while solid curves represent computationally simulated kinetic profiles.

Experimental validation of the rate constants k_3, k_4, k_{-4}, and k_5

To experimentally validate the rate constant k_3, k_4, k_{-4}, and k_5 appearing in the kinetic model of Figure S4, we operated the photochemically triggered transcription system to two auxiliary concentrations of L_1/L_1' (1.6 µM and 0.5 µM). The time-dependent concentration changes of R_1/L_1 were monitored by following the time-dependent fluorescence changes of Cy3 (λ_{em} = 562 nm) using the appropriate calibration curves in Figure S2 and Figure S5B, respectively. Using the Matlab R2019b program, a set of rate constants: $k_3 = 1.0\ \mu M^{-1}\ min^{-1}$, $k_4 = 24.5\ \mu M^{-1}\ min^{-1}$, $k_{-4} = 1.0\ min^{-1}$, $k_5 = 0.1\ \mu M^{-1}\ min^{-1}$ was derived, supporting the computationally simulated rate constant summarized in Table S1. The experimental results fit well to the computationally predicted behaviors of the transcription system.

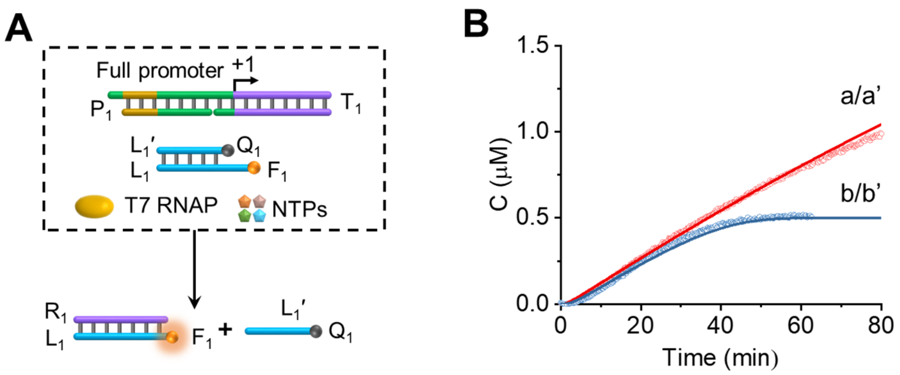

Figure S6. (A) Schematic illustration of fluorescence monitoring of the transcription machinery. (B) The temporal concentrations of the intermediate R_1/L_1 product in the presence of auxiliary concentrations of L_1/L_1': a/a' 1.6 µM, b/b' 0.5 µM. Dots correspond to the experimental data derived by using the appropriate calibration curves shown in Figure S2 an Figure S5B, respectively. Solid curve corresponds to the computationally fitted concentration using the kinetic model presented in Figure S4. Experimental conditions: T_1^* = 0.2 μM, P_1 = 0.2 μM, T7 RNAP = 32 nM, and NTPs = 0.5 mM.

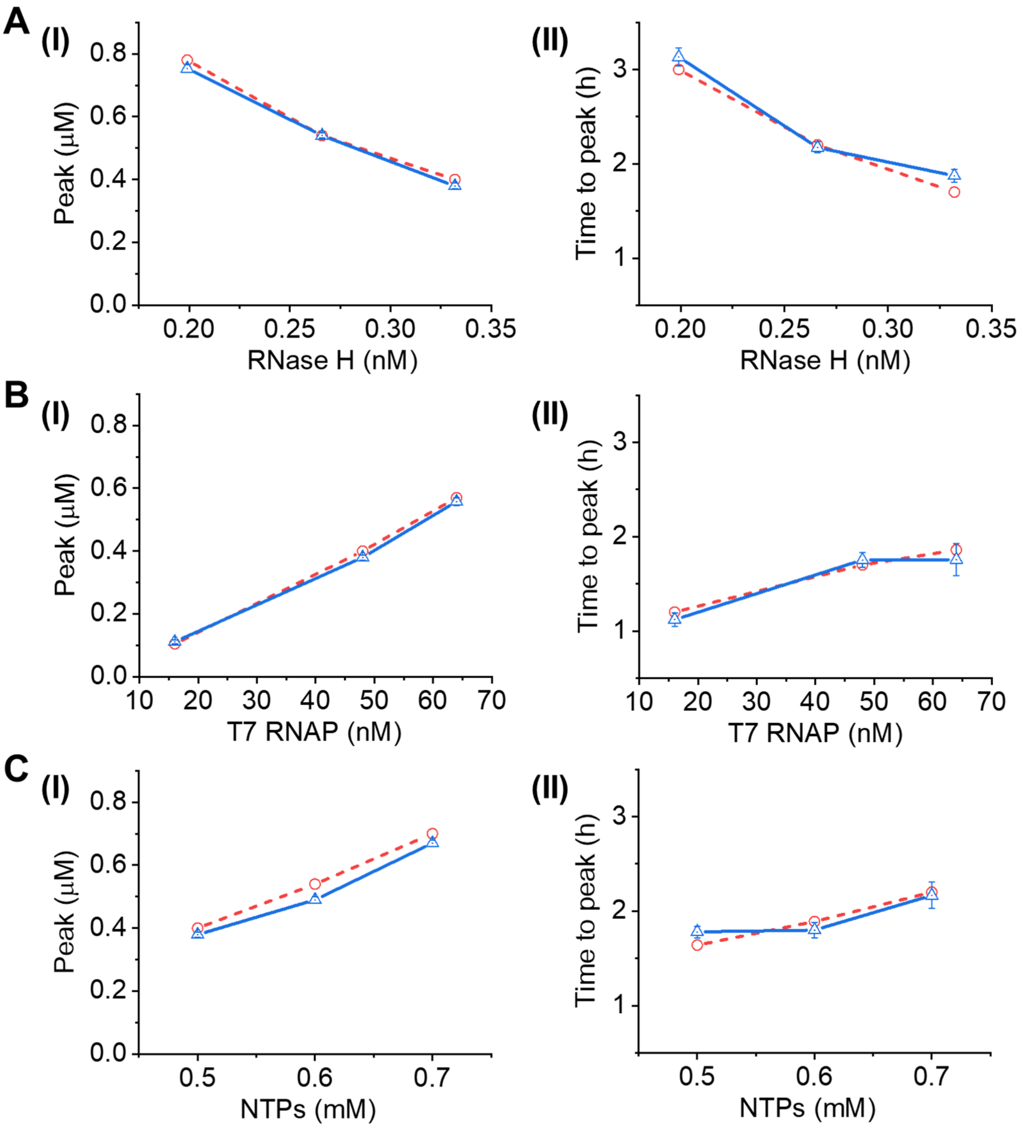

Figure S7. Peak concentration values (panel I) and the time intervals for the emergence of these peak concentrations (panel II) in the presence of variable concentrations of (A) RNase H, (B) T7 RNAP, (C) NTPs. The dashed red curves represent the computationally simulated and predicted results from Figure 1, while the solid blue curves are obtained by experimental results.

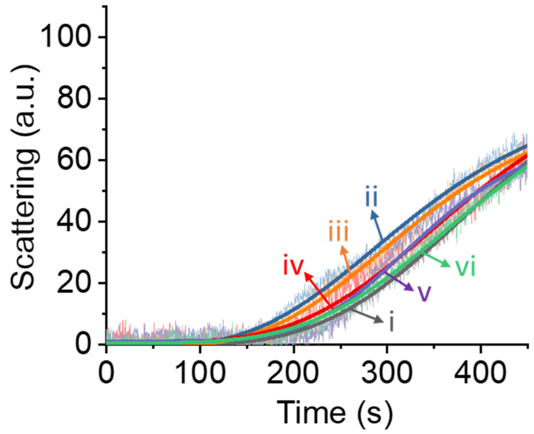

Figure S8. A control study corresponding to Figure 2, showing the temporal light-scattering kinetic profiles of non-illuminated, photocaged reaction samples withdrawn at time intervals: (i) 0, (ii) 2, (iii) 4, (iv) 6, (v) 8, and (vi) 11 hours.

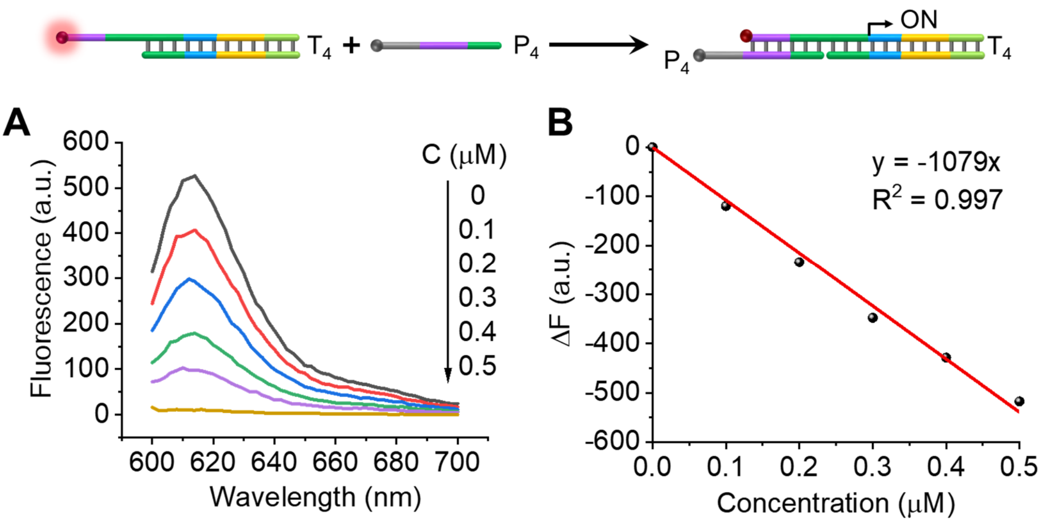

Figure S9. (A) The fluorescence spectra of TexasRed-labeled T_4 (0.5 μM) upon the addition of variable concentrations of Iowa Black®RQ-labeled P_4, leading to the formation of P_4/T_4 and a decrease in the fluorescence intensity (λ_{ex} = 594 nm). (B) The derived calibration curve relating the fluorescence intensity decreases (λ_{em} = 614 nm) of to the concentrations of P_4/T_4.

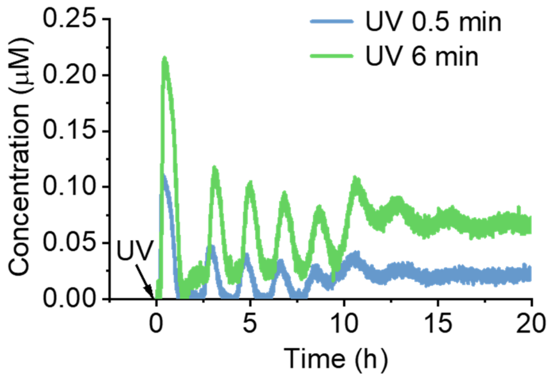

Figure S10. Oscillatory-modulated concentrations of P_4/T_4 generated by the transcripiton circuit over a peirod of 20 hours, resulting from the photochemical uncaging of template T_3* (70 nM) with UV illumination for 0.5 min and 6 min, respectively.

Kinetic equations of photo-activated, oscillatory-modulated transcription circuit:

(1) $T_3 + P_3 \xrightarrow{k_1} P_3T_3$
(2) $T_4 + P_4 \xrightarrow{k_2} P_4T_4$
(3) $B_4 + P_4 \xrightarrow{k_3} B_4P_4$
(4) $R_3 + B_4 \xrightarrow{k_4} R_3B_4$
(5) $R_4 + P_3 \xrightarrow{k_5} R_4P_3$
(6) $R_4 + P_3T_3 \xrightarrow{k_6} R_4P_3 + T_3$
(7) $B_4 + P_4T_4 \xrightarrow{k_7} B_4P_4 + T_4$
(8) $R_3 + B_4P_4 \xrightarrow{k_8} R_3B_4 + P_4$
(9) $P_3T_3 + \text{RNAP} \underset{k_{-9}}{\overset{k_9}{\rightleftarrows}} P_3T_3\bullet\text{RNAP}$
(10) $P_3T_3\bullet\text{RNAP} \xrightarrow{k_{10}} \text{RNAP} + P_3T_3 + R_3$
(11) $P_4T_4 + \text{RNAP} \underset{k_{-11}}{\overset{k_{11}}{\rightleftarrows}} P_4T_4\bullet\text{RNAP}$
(12) $P_4T_4\bullet\text{RNAP} \xrightarrow{k_{12}} \text{RNAP} + P_4T_4 + R_4$
(13) $R_3B_4 + \text{RNaseH} \underset{k_{-13}}{\overset{k_{13}}{\rightleftarrows}} R_3B_4\bullet\text{RNaseH}$
(14) $R_3B_4\bullet\text{RNaseH} \xrightarrow{k_{14}} \text{RNaseH} + B_4$
(15) $R_4P_3 + \text{RNaseH} \underset{k_{-15}}{\overset{k_{15}}{\rightleftarrows}} R_4P_3\bullet\text{RNaseH}$
(16) $R_4P_3\bullet\text{RNaseH} \xrightarrow{k_{16}} \text{RNaseH} + P_3$

Derivatives:

$$\frac{dT_3}{dt} = -k_1[T_3][P_3] + k_6[P_3T_3][R_4]$$

$$\frac{dP_3}{dt} = -k_1[T_3][P_3] - k_5[R_4][P_3] + k_{16}[R_4P_3\bullet\text{RNaseH}]$$

$$\frac{dP_3T_3}{dt} = k_1[T_3][P_3] - k_6[P_3T_3][R_4] - k_9[P_3T_3][\text{RNAP}] + k_{-9}[P_3T_3\bullet\text{RNAP}] + k_{10}[P_3T_3\bullet\text{RNAP}]$$

$$\frac{dT_4}{dt} = -k_2[T_4][P_4] + k_7[P_4T_4][B_4]$$

$$\frac{dP_4}{dt} = -k_2[T_4][P_4] - k_3[B_4][P_4] + k_8[R_3][B_4P_4]$$

$$\frac{dP_4T_4}{dt} = k_2[T_4][P_4] - k_7[B_4][P_4T_4] - k_{11}[P_4T_4][\text{RNAP}] + k_{-11}[P_4T_4\bullet\text{RNAP}] + k_{12}[P_4T_4\bullet\text{RNAP}]$$

$$\frac{dB_4}{dt} = -k_3[B_4][P_4] - k_4[B_4][R_3] - k_7[B_4][P_4T_4] + k_{14}[R_3B_4\bullet\text{RNaseH}]$$

$$\frac{dB_4P_4}{dt} = k_3[B_4][P_4] + k_7[B_4][P_4T_4] - k_8[R_3][B_4P_4]$$

$$\frac{dR_3}{dt} = -k_4[B_4][R_3] - k_8[R_3][B_4P_4] + k_{10}[P_3T_3\bullet\text{RNAP}]$$

$$\frac{dR_3B_4}{dt} = k_4[R_3][B_4] + k_8[R_3][B_4P_4] - k_{13}[R_3B_4][\text{RNaseH}] + k_{-13}[R_3B_4\bullet\text{RNaseH}]$$

$$\frac{dR_4}{dt} = -k_5[R_4][P_3] - k_6[R_4][P_3T_3] + k_{12}[P_4T_4\bullet\text{RNAP}]$$

$$\frac{dR_4P_3}{dt} = k_5[R_4][P_3] + k_6[R_4][P_3T_3] - k_{15}[R_4P_3][\text{RNaseH}] + k_{-15}[R_4P_3\bullet\text{RNaseH}]$$

$$\frac{d\text{RNAP}}{dt} = -k_9[P_3T_3][\text{RNAP}] + k_{-9}[P_3T_3\bullet\text{RNAP}] + k_{10}[P_3T_3\bullet\text{RNAP}] - k_{11}[P_4T_4][\text{RNAP}] + k_{-11}[P_4T_4\bullet\text{RNAP}] + k_{12}[P_4T_4\bullet\text{RNAP}]$$

$$\frac{dP_3T_3\bullet\text{RNAP}}{dt} = k_9[P_3T_3][\text{RNAP}] - k_{-9}[P_3T_3\bullet\text{RNAP}] - k_{10}[P_3T_3\bullet\text{RNAP}]$$

$$\frac{dP_4T_4\bullet\text{RNAP}}{dt} = k_{11}[P_4T_4][\text{RNAP}] - k_{-11}[P_4T_4\bullet\text{RNAP}] - k_{12}[P_4T_4\bullet\text{RNAP}]$$

$$\frac{d\text{RNaseH}}{dt} = k_{-13}[R_3B_4\bullet\text{RNaseH}] - k_{13}[R_3B_4][\text{RNaseH}] + k_{14}[R_3B_4\bullet\text{RNaseH}] - k_{15}[R_4P_3][\text{RNaseH}] + k_{-15}[R_4P_3\bullet\text{RNaseH}] + k_{16}[R_4P_3\bullet\text{RNaseH}]$$

$$\frac{dR_3B_4\bullet\text{RNaseH}}{dt} = k_{13}[R_3B_4][\text{RNaseH}] - k_{-13}[R_3B_4\bullet\text{RNaseH}] - k_{14}[R_3B_4\bullet\text{RNaseH}]$$

$$\frac{dR_4P_3\bullet\text{RNaseH}}{dt} = k_{15}[R_4P_3][\text{RNaseH}] - k_{-15}[R_4P_3\bullet\text{RNaseH}] - k_{16}[R_4P_3\bullet\text{RNaseH}]$$

Figure S11. Computational simulation of the photo-activated, oscillatory-modulated transcription circuit. The kinetic scheme of the sub-reactions associated with the time-dependent concentration changes are summarized in the above equations. Knowing the time-dependent concentration changes of the P_4/T_4 during transcription, we computationally simulated the system by using Matlab R2019b. Initial concentrations of the condition: T_3* = 150 nM, P_3 = 1.5 µM, B_4 = 1.5 µM, P_4 = 0.5 µM, T_4 = 0.5 µM, T7 RNAP = 128 nM, RNase H = 1.00 nM, and NTPs = 7.5 mM at 35 °C. Note that, since the concentration of NTPs is in excess, we assume that, within the 10-hour time scale, the changes in NTP concentration are minimal and do not affect the transcription kinetics.

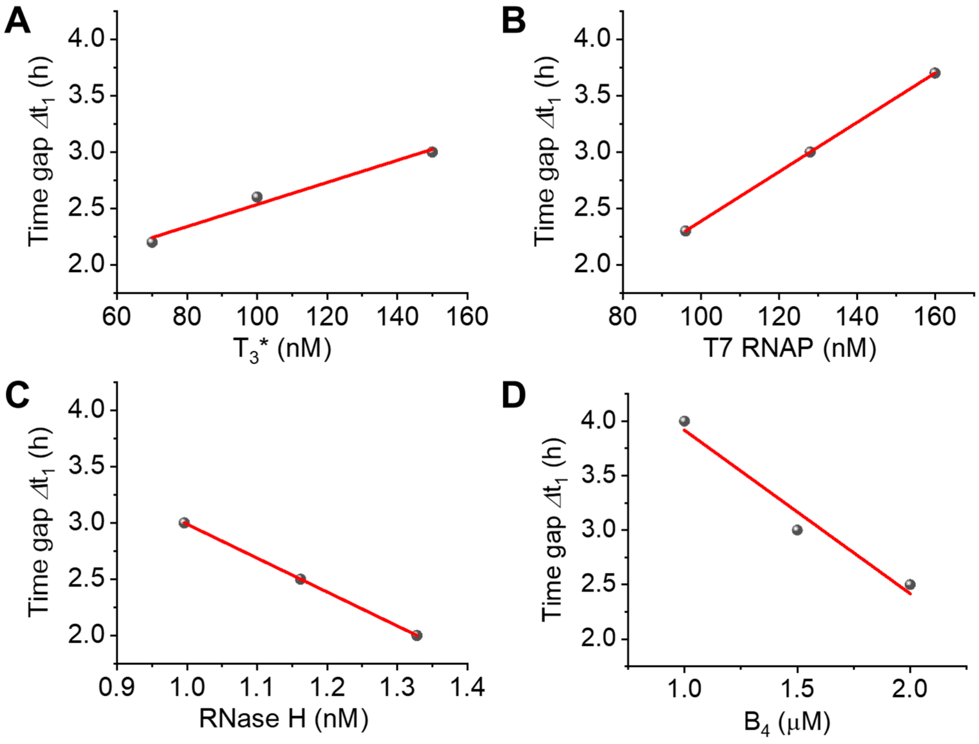

Figure S12. The computationally simulated time gaps between the first and second oscillating peaks of the photo-activated, oscillatory-modulated transcription circuit in the presence of variable concentrations of (A)T_3*, (B) T7 RNAP, (C) RNase H, and (D) B_4.

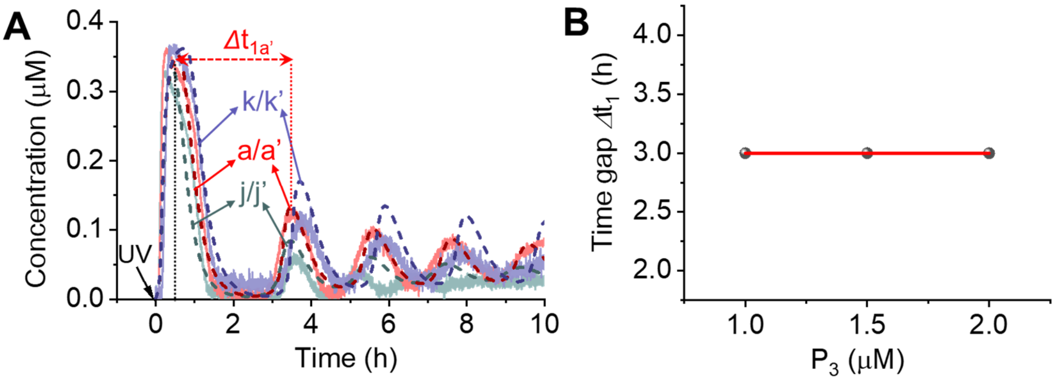

Figure S13. (A) Temporally oscillatory-modulated concentrations of P_4/T_4 generated by the circuit in the presence of different concentrations of P_3: (a/a′) 1.5 μM, (j/j′) 1.0 μM, and (k/k′) 2.0 μM. Dashed curves are computationally predicted curves, and solid curves represent experimentally validated results. Other experimental conditions for these curves are similar to those of curve a. (B) The computationally simulated time gaps between the first and second oscillating peaks of the photo-activated, oscillatory-modulated transcription circuit in the presence of variable concentrations of P_3.

Effect of simultaneous concentration changes of B_4 and RNase H on the photo-triggered dynamic transient oscillation of the system displayed in Figure 3

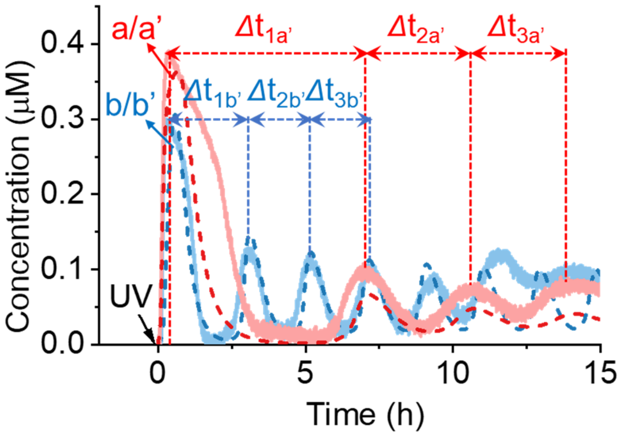

Figure S14. Temporally oscillatory-modulated concentrations of P_4/T_4 generated by the photo-triggered transcripiton circuit shown in Figure 3. Dashed curves are computationally predicted curves, and solid curves represent experimentally validated results. Curves a/a': B_4 = 1.0 µM, RNase H = 0.66 nM. Curves b/b': B_4 = 1.5 µM, RNase H = 1.00 nM. Other experimental conditions: T_3^* = 100 nM, P_3 = 1.5 µM, P_4 = 0.5 µM, T_4 = 0.5 µM, T7 RNAP = 128 nM, and NTPs = 7.5 mM at 35 °C, UV illumination (365 nm, 6 min). $\Delta t_{1a'}$ = 6.6 h, $\Delta t_{2a'}$ = 3.5 h, $\Delta t_{3a'}$ = 3.3 h. $\Delta t_{1b'}$ = 2.6 h, $\Delta t_{2b'}$ = 2.0 h, $\Delta t_{3b'}$ = 2.0 h.

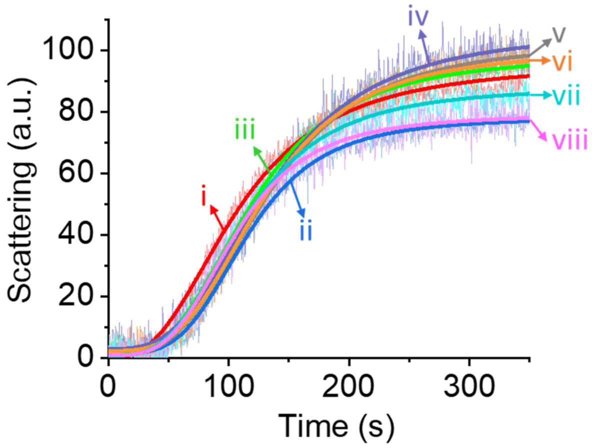

Figure S15. A control study corresponding to Figure 5, showing the temporal light-scattering kinetic profiles of non-illuminated, photocaged reaction samples withdrawn at different time intervals: (i) 0, (ii) 20, (iii) 100, (iv) 200, (v) 300, (vi) 400, (vii) 500, and (viii) 600 min.

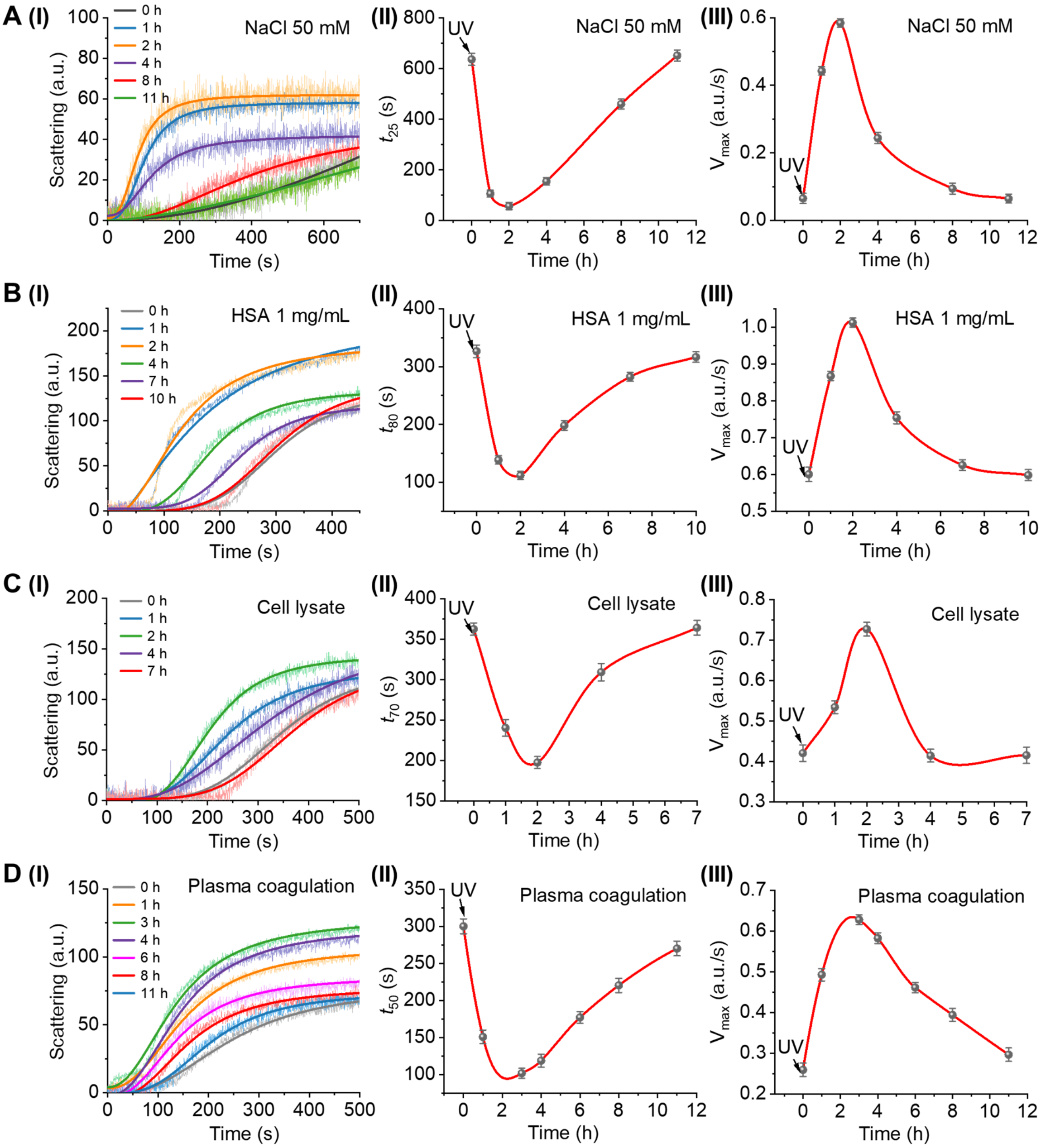

Figure S16. Effects of auxiliary perturbances on the photo-triggered transient transcription machinery-guided coagulation of fibrinogen to fibrin according to Figure 2A: (A) In the present of NaCl, 50 mM; (B) In the presence of HSA, 1 mg/mL; (C) In the presence of cell lysate; (D) Coagulation of human plasma. Panels I display the temporal light-scattering kinetic profiles corresponding to the fibrinogenesis processes in samples withdrawn from the reaction mixtures. Panels II present the temporally oscillatory-modulated t values reaching the respective scattering intensity thresholds. Panels III display the temporally oscillatory-modulated V_{max} values.

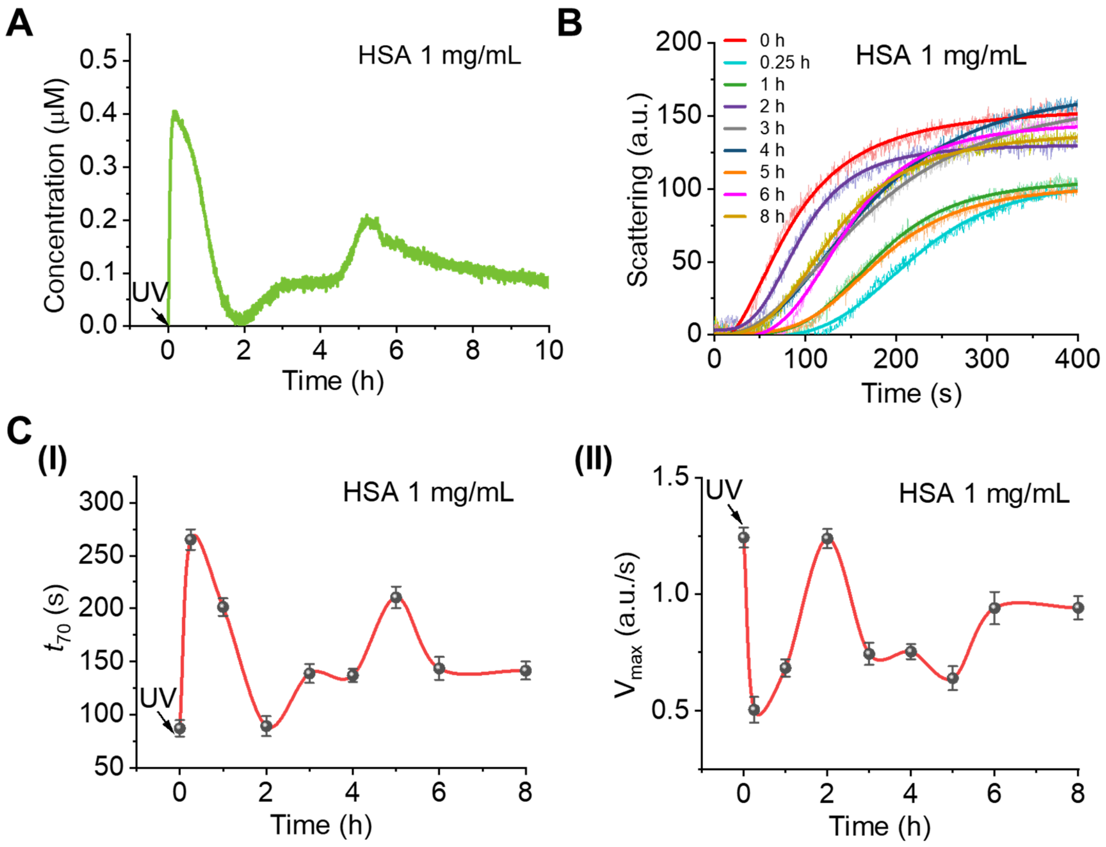

Figure S17. (A) Effect of HSA (1 mg/mL) on the photo-triggered oscillatory transient transcription circuit depicted in Figure 3. (B) Temporal light-scattering kinetic profiles corresponding to the photo-triggered oscillatory transcription circuit operating in the presence of HSA (1 mg/mL), inducing the coagulation of fibrinogen to fibrin at time intervals. (C) Analysis of the temporal light-scattering kinetic profiles shown in (B): panel I — temporally oscillatory-modulated t_{70} values, panel II — temporally oscillatory-modulated V_{max} values.

Table S1. Rate constants derived from the computational simulation of the photochemically triggered transient transcription machinery shown in Figure S4 (T = 35 °C).

k_1	5.13 μM^{-1} min^{-1}	k_2	26.0 μM^{-1} min^{-1}	k_3	1.00 μM^{-1} min^{-1}
k_4	24.5 μM^{-1} min^{-1}	k_{-4}	1.00 min^{-1}	k_5	0.100 μM^{-1} min^{-1}
k_6	160 μM^{-1} min^{-1}	k_{-6}	10.0 min^{-1}	k_7	81.7 min^{-1}

Table S2. Rate constants derived from the computational simulation of the photo-activated oscillatory-modulated transcription circuit shown in Figure S11 (T = 35 °C).

k_1	5.13 μM^{-1} min^{-1}	k_2	0.80 μM^{-1} min^{-1}	k_3	29.0 μM^{-1} min^{-1}
k_4	0.100 μM^{-1} min^{-1}	k_5	1.00 μM^{-1} min^{-1}	k_6	0.569 μM^{-1} min^{-1}
k_7	0.085 μM^{-1} min^{-1}	k_8	10.0 μM^{-1} min^{-1}	k_9	24.5 μM^{-1} min^{-1}
k_{-9}	3.36 min^{-1}	k_{10}	10.0 min^{-1}	k_{11}	24.5 μM^{-1} min^{-1}
k_{-11}	1.61 min^{-1}	k_{12}	2.50 min^{-1}	k_{13}	160 μM^{-1} min^{-1}
k_{-13}	10.0 min^{-1}	k_{14}	180 min^{-1}	k_{15}	160 μM^{-1} min^{-1}
k_{-15}	1.00 min^{-1}	k_{16}	137 min^{-1}		

Supplemental References

(1) Dong, J.; Willner, I. Dynamic transcription machineries guide the synthesis of temporally operating DNAzymes, gated and cascaded DNAzyme catalysis. *ACS Nano* **2023**, *17*, 687–696.
